# Conductive Polymeric-Based Electroactive Scaffolds for Tissue Engineering Applications: Current Progress and Challenges from Biomaterials and Manufacturing Perspectives

**DOI:** 10.3390/ijms222111543

**Published:** 2021-10-26

**Authors:** Maradhana Agung Marsudi, Ridhola Tri Ariski, Arie Wibowo, Glen Cooper, Anggraini Barlian, Riska Rachmantyo, Paulo J. D. S. Bartolo

**Affiliations:** 1 Materials Science and Engineering Research Group, Faculty of Mechanical and Aerospace Engineering, Institut Teknologi Bandung, Jl. Ganesha 10, Bandung 40132, West Java, Indonesia; maradhanaa@alumni.itb.ac.id (M.A.M.); ariskiridh@gmail.com (R.T.A.); rachmantyo@material.itb.ac.id (R.R.); 2 Research Center for Nanoscience and Nanotechnology, Institut Teknologi Bandung, Jl. Ganesha 10, Bandung 40132, West Java, Indonesia; 3 Department of Mechanical, Aerospace, and Civil Engineering, University of Manchester, Manchester M13 9PL, UK; glen.cooper@manchester.ac.uk (G.C.); paulojorge.dasilvabartolo@manchester.ac.uk (P.J.D.S.B.); 4 School of Life Science & Technology, Institut Teknologi Bandung, Jl. Ganesha 10, Bandung 40132, West Java, Indonesia; aang@sith.itb.ac.id

**Keywords:** additive manufacturing, bone, cardiac, conductive polymers, electroactive scaffold, muscle, nerve, skin, tissue engineering

## Abstract

The practice of combining external stimulation therapy alongside stimuli-responsive bio-scaffolds has shown massive potential for tissue engineering applications. One promising example is the combination of electrical stimulation (ES) and electroactive scaffolds because ES could enhance cell adhesion and proliferation as well as modulating cellular specialization. Even though electroactive scaffolds have the potential to revolutionize the field of tissue engineering due to their ability to distribute ES directly to the target tissues, the development of effective electroactive scaffolds with specific properties remains a major issue in their practical uses. Conductive polymers (CPs) offer ease of modification that allows for tailoring the scaffold’s various properties, making them an attractive option for conductive component in electroactive scaffolds. This review provides an up-to-date narrative of the progress of CPs-based electroactive scaffolds and the challenge of their use in various tissue engineering applications from biomaterials perspectives. The general issues with CP-based scaffolds relevant to its application as electroactive scaffolds were discussed, followed by a more specific discussion in their applications for specific tissues, including bone, nerve, skin, skeletal muscle and cardiac muscle scaffolds. Furthermore, this review also highlighted the importance of the manufacturing process relative to the scaffold’s performance, with particular emphasis on additive manufacturing, and various strategies to overcome the CPs’ limitations in the development of electroactive scaffolds.

## 1. Introduction

With a strong growth in the field of tissue engineering over the last few decades, the standard for an effective bio-scaffold, which holds an integral role in the process of tissue repair, has also risen over time. The new generation of smart bio-scaffolds are not only able to act as a media or matrix for cellular adhesion, but are also able to control the cellular activities, support cellular proliferation process and promote new tissue specialization [1,2]. In this context, natural-based (e.g., chitosan, gelatin, alginate) and synthetic-based polymers (e.g., polylactide, polycaprolactone, polyvinyl alcohol) are the current dominant class of material for bio-scaffold in tissue engineering due to their processability, biocompatibility, possible biodegradability and similar mechanical properties to most natural tissues [3,4,5,6,7]. Nevertheless, these polymers mostly only act as a passive scaffold to temporarily support the biological structure while waiting for the tissues to naturally recover, without being able to actively provide any support towards cell proliferation and guide cell differentiation.

Bioactivity in a scaffold can be imbued by several approaches. A common approach to accelerate the tissue regenerations rate is by utilization of materials that are widely found in the natural tissue (e.g., collagen, which are commonly found in the extracellular matrix (ECM)) [8]. In specific cases such as bone tissue engineering, hydroxyapatite is a commonly used additive to enhance biomineralization and promote osteogenesis [9]. However, natural polymers have a wide range of natural viability, and their structures are more complex than synthetic polymers, making it difficult to tailor their properties to be used as bio-scaffolds, while the introduction of additives are tissue-specific and may not be an applicable strategy for all tissues. On the other hand, synthetic polymers are much more adjustable in terms of structure and properties. Scaffolds with similar properties (e.g., mechanical, electrical, physiochemical) to the native tissues will result in an enhanced rate of recovery and promote specialization, as the scaffold could provide signals and cues to properly guide new tissue growth [1,2,3,4,5,6,7,8,9,10,11]. These strategies are well-established and are generally regarded as a safe method to imbue some degree of bioactivity to the scaffold. However, the rate of tissue recovery in these ‘passive’ scaffolds are often unsatisfactory, with many studies reporting up to several weeks or even months until the tissues are considered to be fully recovered [12,13].

**Figure 1:**
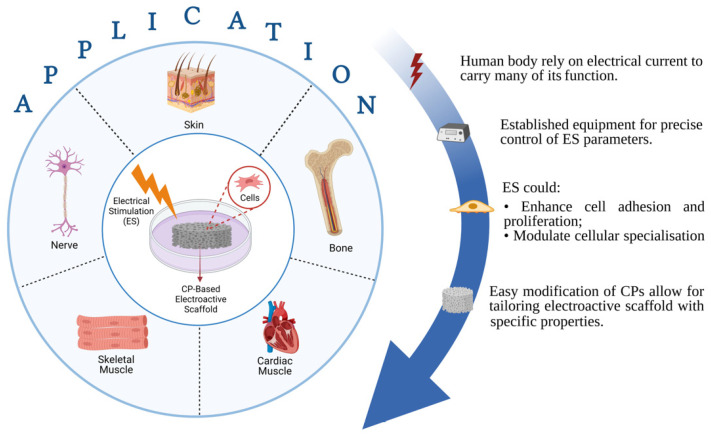
Illustration about advantages of conductive polymeric (CP)-based electroactive scaffold and their electrical stimulation for various tissue engineering applications.

A more ‘aggressive’ approach to further enhance bioactivity may seek to combine the polymeric scaffold with another supporting factor that can enhance the rate of new tissue formation. One commonly used strategy is to incorporate growth factors (e.g., vascular endothelial growth factor (VEGF) [14], bone morphogenetic protein 2 (BMP-2) [15], etc.) into the scaffold ex vivo prior to implantation, which will then be released in a controlled manner in vivo [16,17]. Although phase I trials generally reported promising results, practical application of these approaches are currently obstructed by the strict regulatory approval, as there have been several studies that point to the unwanted formation of dormant tumors when sufficiently large quantities of growth factors were administered [18,19]. Another approach relies on utilizing external stimulation therapy along with stimuli-responsive scaffold as a means of providing cues to guide cellular specialization and promote tissue maturation. These external stimuli may vary from mechanical and biochemical [20,21], magnetic [22], ultrasound [23], and electrical stimulation [24,25], among others. In particular, the usage of electrical stimulation (ES) therapy alongside electroactive scaffolds is regarded as one attractive and promising approach, as it has the established equipment to allow precise control in terms of various therapeutical parameters (e.g., voltage magnitude, duration and interval between pulses), and ES in itself (without accompanied by electroactive scaffold) has been widely used in clinical practices [26,27]. Considering the fact that the human body relies on electrical current to carry many of its functions, the utilization of electroactive scaffolds alone—even without ES—is able to imbue bioactivity, where the conductive scaffold could provide cues to guide tissue formation due to the presence of endogenous electrical fields in the tissue microenvironments [28]. Nevertheless, ES can be used in conjunction with electroactive scaffolds to assist recovery by enhancing cell adhesion and proliferation as well as modulating cellular specialization, and the success has been reported in several tissue engineering applications including bone [29,30], skin [31], neural [32,33], skeletal muscle [34] and cardiac muscle tissues [35,36] (Figure 1). ES are shown to be favorable towards tissue formation, does not negatively affect cell viability in a significant manner, and is considered to be a safe option (potentially as safe as other external stimulation therapies), with no reports regarding harmful long-term effects have been reported so far [37,38,39]. However, the statement is valid only when the ES is operated within the appropriate parameters alongside the scaffold with conductivity in the range of the native tissues, thus extra caution must be made to ensure that all the correct parameters and properties are in place. Overly conductive scaffolds could trigger cell death due to the cell receiving voltage higher than its survival threshold, whereas insufficiently conductive (overly resistive) scaffold may cause the scaffold to be overheated as a result of the applied voltage, which could cause cell death due to protein denaturation [39]. With that in mind, it is imperative that the scaffold’s conductivity must be tailored to be in the range of the native tissues to ensure the biocompatibility of ES.

In fabricating an electroactive scaffold, electrically conductive materials including carbon-based materials (carbon nanotubes [40,41], graphene [42]), metallic nanostructures [29,43], and conducting polymers (CPs) [44,45] are usually mixed in a composite system alongside the previously mentioned biocompatible polymers. Among these materials, CPs have gained emerging attention particularly due to their easy synthesis and modification that allow for tailoring electroactive scaffold with specific properties (Figure 1) [2]. CPs such as polypyrrole (PPy), polyaniline (PANI), and polythiophene (PTh) derivatives are inherently conductive due to the presence of conjugated chains containing localized carbon-carbon single bonds and less localized carbon-carbon double bonds in their backbone. The electrons are able to move along the polymer chain due to the p-orbitals overlap in the double bonds, thus giving the electron greater mobility between atoms [46]. Their conductivity can be further improved by introducing dopant ions which can disrupt the CP backbone by introducing charge carrier and transfer charge along the polymer, thus a given CP can have a large range of conductivity similar to semiconductors or even metallic conductors [47]. This widely tunable conductivity, alongside the previously listed advantages, have made CPs widely used materials in tissue engineering.

Despite all the promises and potential offered by ES and CP-based scaffolds, its practical application is still largely limited by its unoptimized properties, many of which are caused by the properties of CPs themselves. In this review, the latest (2015–2021) application of CPs-based electroactive scaffolds and their improvement strategies to meet the requirement in biomedical application is thoroughly discussed. This review will start with addressing and discussing the issues that are commonly experienced in CP-based electroactive scaffolds in tissue engineering, including its mechanical properties, biocompatibility, hydrophilicity, and biodegradability. Then, it will be followed by highlighting more specific problems pertinent to each individual tissues including bone, nerve, skin, skeletal and cardiac muscle, each having different and specific requirements. In addition, this review will also highlight the importance of manufacturing process relative to the scaffold’s performance, with particular emphasis on additive manufacturing.

## 2. General Improvement Strategies for CP-Based Electroactive Scaffolds

**Figure 2:**
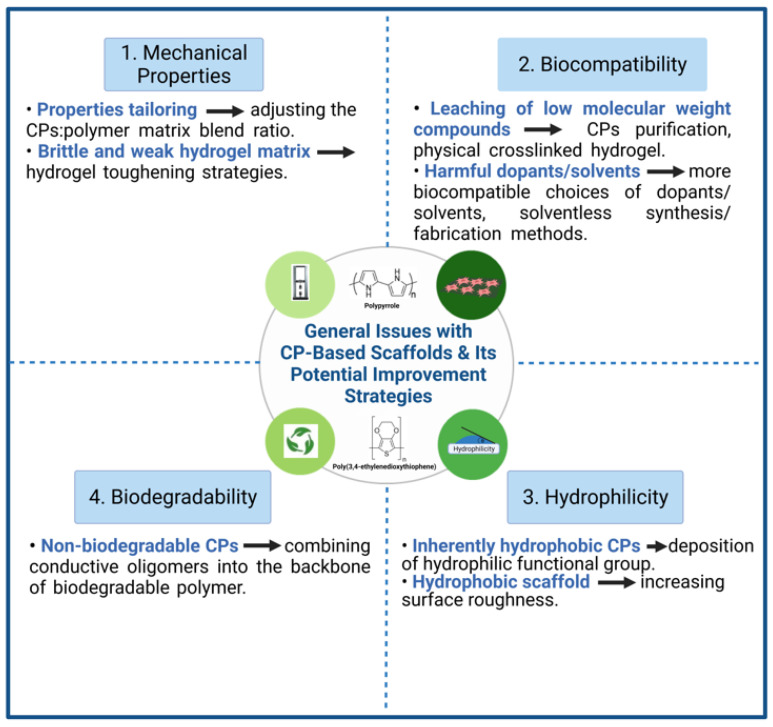
General issues with CP-based scaffolds and its potential improvement strategies.

Even though CPs have great potential in tissue engineering applications, CPs are still haunted with several weaknesses and limitations pertinent to their intrinsic properties. Although the main benefit of adding CPs into a scaffold is to imbue electroactive properties, for an in vivo implant, CP-based scaffolds must also be mechanically stable, biocompatible and bioactive. The concept of using pure CPs alongside ES in tissue engineering have been reported and are favorable for cell maturation. Nevertheless, they are restricted in the form of thin films with no data regarding its mechanical performances and biocompatibility, thus making pure CPs not applicable as in vivo implant [48,49]. Discussions within this review will be aimed at CP-polymer composite, as it is by far the most common class of CP-based electroactive scaffold. This section will discuss the inherent weaknesses of CP-based scaffolds that may hinder its application in tissue engineering and electroactive scaffolds in general, as well as highlighting some strategies that have been, or could have the potential to be employed to resolve these issues (summarized in Figure 2).

### 2.1. Mechanical Properties

CPs in themselves are known to be very brittle, therefore making it difficult to fabricate a conductive scaffold using high concentration of CPs [2]. The common strategy to overcome its inherent brittleness is to blend together small but adequate quantity of CPs—just enough to mimic the natural tissue’s conductivity—alongside non-conductive polymers (e.g., PLA, PCL, chitosan, etc.) or hydrogels that are less brittle than the CPs as matrix, creating a composite. In turn, the introduction of CPs can help to improve the modest mechanical strength and Young’s modulus of the non-conductive polymer, which are often too low for practical use as bio-scaffolds. In this sense, the CPs can be seen as filler particles (i.e., load-carrying medium) in a composite that will help strengthen the non-conductive polymer matrix (i.e., load-transporting medium), provided that there are sufficient interaction forces between the CP’s and the polymer matrix’s interface.

**Figure 3:**
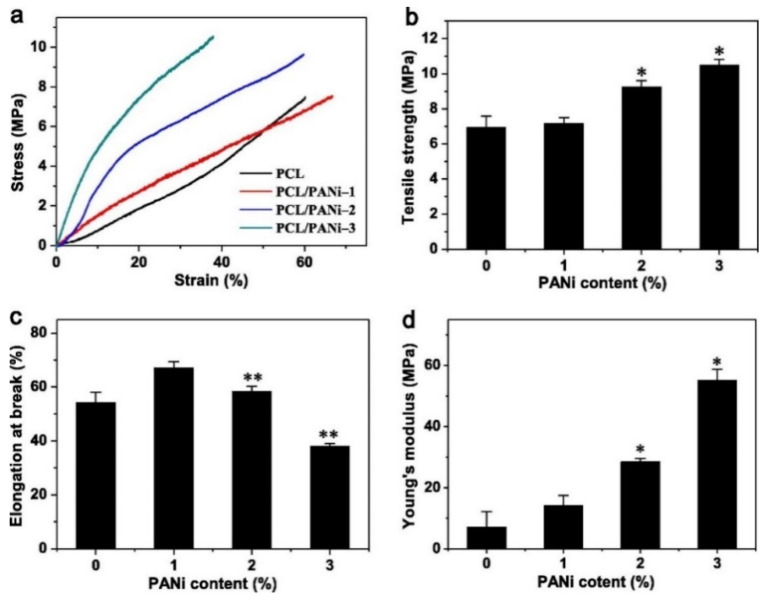
Effect of incorporating PANI into PCL nanofibrous scaffold to its mechanical properties. (**a**) Stress-strain curve, (**b**) Tensile strength, (**c**) Elongation at break, and (**d**) Young’s modulus. * Significantly different from pure PCL (*p* < 0.05, *n* = 5); ** Significantly different from PCL-PANI-1 (*p* < 0.05, *n* = 5). Reproduced with permission from [51]. Copyright (2013) Elsevier.

In film and fiber-based composite scaffolds, the main challenge often comes in the form of tailoring the right mechanical properties for specific tissues. Sometimes, an increase in stiffness is needed, while other times it needs to be decreased. This issue can be addressed by adjusting the ratio between CPs and its polymer matrix. For instance, blending of PEDOT:PSS with chitosan and polyvinyl alcohol (PVA) via solution electrospinning was able to tune both the stiffness and tensile strength of the nanofibrous scaffold to better match the mechanical properties of cardiac muscle tissues [50]. When used as the matrix, the chitosan/PVA blend used in this study is able to significantly reduce the overly high stiffness and increase the elongation at break of the PEDOT:PSS. An increase in Young’s modulus, strength and toughness is directly proportional to the PEDOT:PSS content ratio (up to 1 wt%). This was mainly attributed to the reduction in fiber diameter caused by the addition of PEDOT:PSS, which in turn will result in higher crystallinity and more aligned molecular orientation. Hydrogen bond was also introduced as a result of the interaction between the OH groups in PVA and chitosan with the SO_3_^−^ groups in PSS, which may also contribute to the enhanced mechanical properties. A similar trend was also observed in PCL/PANI nanofiber scaffold, reporting increasing tensile strength and significantly increased Young’s modulus (almost 8-fold at 3% wt PANI) as the content of PANI goes up, although elongation at break may be compromised (Figure 3) [51]. This phenomenon is also observed in 3D architecture, as is seen in our previous work which indicated that increasing the weight percentage of PANI in PCL/PANI bone scaffold can help increase the Young’s modulus and compressive strength of the scaffold [44]. At 2 wt% PANI, the scaffold was 28% stiffer than pure PCL scaffold from 64.43 MPa to 82.61 MPa, making it mechanically more suitable for application as a cancellous bone scaffold. It should be noted that not every addition of CPs into non-conductive polymer matrix resulted in increased mechanical properties, as was demonstrated in silk fibroin scaffold [52] or in chitosan/collagen scaffold [53] which experienced reduction in Young’s modulus and tensile strength when PPy was added. Some plausible explanations are attributed to the fragility of PPy, non-homogeneous CP distribution, or the lack of strong interfacial interaction between PPy and the matrix (thus the CP particles are viewed as holes/porosities rather than strengthening filler particles), but these claims are rarely backed up by experimental results, and the exact reasons thus far are still inconclusive.

On the other hand, the problems with hydrogel-based conductive scaffolds in terms of mechanical properties is more one-directional compared to films and fiber-scaffolds. While CP-based films and nanofibers scaffolds can possess both overly high or overly low stiffness as previously discussed, CP-based hydrogels are almost always on the weaker side in terms of the mechanical properties due to its highly porous structure and hydrated nature. This often becomes a hindrance for in vivo applications that experiences substantial mechanical loading (e.g., cartilage, skeletal and cardiac muscle). This issue is both experienced in conductive hydrogel composite, as well as in films grown on top of hydrogel substrate (i.e., layered structure). For instance, PANI/gelatin gum hydrogel as shown in the work of Shrisuk et al. exhibits a compressive modulus of ~30 kPa and relatively poor elastic recovery capability, which may limit its practical application as in vivo muscle implant despite its excellent ability in promoting differentiation of myoblasts into myotubes [54]. Simply adding higher concentration of CPs may not solve the whole problem, as there is a certain limit of how high the concentration can go before excessive CP loading may cause a decrease in mechanical properties due to heterogeneous composite formation, as well as other issues such as diminished biocompatibility [55]. Therefore, in this case, the mechanical properties of the matrix itself may be improved.

To improve the hydrogel’s mechanical properties (and thus the overall scaffold), tough structures such as double network (DN) or triple network may be employed [56,57]. For layered architecture, PEDOT/polyurethane elastic electrode was electropolymerized on top of DN gel substrate, where the DN gel possess extremely high mechanical integrity when compared to conventional single network hydrogels [58]. The DN gel, which in this case consists of poly(2-acrylamido-2-methylpropanesulfonic acid) and poly(acrylamide) (PAMPS-PAAm), has been used in various biomedical applications that requires exceptional mechanical properties [59,60,61]. Without compromising conductivity and biocompatibility, the utilization of double network structure managed to significantly improve the scaffold’s durability, which remains electrically stable after 100 repeated cycles of bending and stretching. DN gel can also be fabricated in the form of CP-hydrogel composite [62,63]. Darabi et al. fabricated PPy-grafted chitosan as its first network, and poly(acrylic acid) (PAA) as its second network [62]. Reversible ionic interaction between carboxylic group in PAA and NH group in PPy allows the scaffold to possess self-healing property. At its highest crosslinking density, the DN gel possess very high compressive modulus (up to 800 kPa). However, addition of PPy can actually lower the modulus since the hydrogen and ionic bonding between PPy and the gel is weaker than the covalent bonds present in the DN gel, and the crosslinking density can be reduced and tailored to better match the modulus of the replaced tissue.

### 2.2. Biocompatibility

Currently, there are conflicting reports regarding the biocompatibility of CPs. For example, although reported to be generally safe and biocompatible in low concentration, the usage of PANI in high concentration are reported to be cytotoxic and even promote chronic inflammation when implanted in vivo [44,64,65]. Contrary to popular beliefs, PPy which is often viewed as a CP with more favorable biocompatibility than PANI does not fare much better in terms of biocompatibility, showing mild to moderate toxicity against common fibroblasts NIH/3T3 cells at a solution concentration of 10%, which is similar in performance with PANI when compared side-by-side [66]. However, since most of these CPs are fully insoluble in aqueous condition, it is likely that the one causing biocompatibility issues are not the materials of the CPs themselves. Further supporting this hypothesis is the fact that even though PEDOT:PSS is dispersible in water and can be formulated as water-soluble molecule, it also reported excellent biocompatibility [67]. Therefore, cytotoxicity has been related to the leaching of low molecular weight compounds found in the CPs, which can be in the form of its leftover/unpolymerized oligomers, or leftover acids that helps form the CPs during the synthesis process [68]. Size and shapes of the CPs in the composite may also plays a part in determining the overall scaffold’s cytotoxicity [66]. The insolubility and hydrophobicity of CPs can also trigger an immune response and subsequently cause inflammation, but discussion in improving the CP’s hydrophilicity will be split into the next part, whereas this part will focus on solving the issues around preventing the low molecular weight compounds to cause further unwanted cytotoxicity.

To remove the unwanted impurities from the CPs, several methods of purifications can be used. Since the transition between PANI base (non-conductive form) and PANI salt (conductive form) is reversible, Humpolicek et al. used a purification method involving cycles of deprotonation of PANI salt and reprotonation of PANI base in order to remove the low molecular weight impurities from the samples as much as possible [69]. The sample which underwent deprotonation and reprotonation reported significantly higher biocompatibility, being able to support cell viability of HaCaT at a value of 0.67 (mild cytotoxicity) compared to untreated samples of the same concentration at 0.40 (severe cytotoxicity), supporting the hypothesis that removal of low molecular weight impurities play a huge part in improving the overall CP-based scaffold’s biocompatibility. Another method of post-synthesis purification in the form of reprecipitation was also used for the removal of residual monomers [70]. In this procedure, the CP is dissolved in a suitable solvent (for PANI, N-methyl pyrrolidone can be used, even though the solubility is not complete), and then added dropwise to a non-solvent, allowing the CP to precipitate while the monomers stay dissolved. The purified sample also shows much higher cell viability, reporting 0.89 (no cytotoxicity) compared to 0.56 (moderate toxicity) of untreated PANI at the concentration of 5%. In this study, the group reported relatively comparable cytotoxicity between globular and nanotubular morphology of PANI. However, another study reported that the size of PPy nanoparticles have a significant effect on the cell viability of human lung fibroblast, where larger particle size will generally result in lower cytotoxicity [71].

Acid doping is a commonly used strategy to oxidize CPs such as PPy and PANI, converting them from its non-conductive form to its conductive form. In this case, dopants are proton donors (p-doping) and are usually strong acids such as hydrochloric acid (HCl) and sulfuric acid (H_2_SO_4_). However, these acids may cause cytotoxicity issues in the cellular environment, especially when not removed properly after synthesis [72]. Thus, biocompatibility of the resulting PANI can be increased by substituting the acids with a more biocompatible acid, as was shown in the work of Daraeinejad and Shabani, who replaced camphorsulfonic acid (CSA) with taurine [73]. Aside from being less toxic than CSA, some studies have also shown that taurine can promote cell proliferation and differentiation in neural tissues, thus making it bioactive [74]. The cellular viability of 3T3 cell is significantly higher in the PANI/poly(ether sulfonate) scaffold treated with taurine (more than 0.80 value after 7 days which indicates no cytotoxicity) compared to the CSA-treated scaffold (below 0.60 value after 7 days, indicating moderate cytotoxicity). Nevertheless, conductivity was compromised as a result of taurine’s shorter molecular chains which leads to better PANI solubility in the composite (0.5 × 10^−5^ S/cm in taurine-treated scaffold compared to 3.7 × 10^−5^ S/cm in CSA-treated scaffold).

Blending CPs with non-conductive biocompatible polymers also serves as a means to enhance the scaffold’s biocompatibility. In fact, biocompatibility is often not an issue when the weight percentage of the CPs is sufficiently low compared to the biocompatible polymer matrix, while adequate conductivity and mechanical properties can usually be achieved even with low CP concentration [44,75]. Nevertheless, mishandling during blending or with the blended materials themselves may possess threat to biocompatibility. As previously stated, leaching of solvents involved during synthesis can impart negative effect on biocompatibility, as the organic solvents used during solvent-based synthesis such as solution electrospinning may potentially be unsafe for biomedical uses (e.g., chloroform, dimethylformamide, etc.) [76,77,78]. Thus, solvent-less methods of blending such as melt electrospinning or melt extrusion additive manufacturing may be employed instead [79]. And although the usage of hydrogel-based scaffolds generally leads to better biocompatibility due to the similarity in the nature of hydrogel and ECM, leaching of unreacted crosslinker monomers may possess unwanted cytotoxic issues. To resolve this, physically crosslinked hydrogels which relies on ionic crosslinking or hydrogen bonding can be chosen as a safer alternative rather than conventional chemically crosslinked hydrogels with covalent bonds [80]. Bi et al. constructed a physically crosslinked chitosan/PVA hydrogel with the aforementioned double network structure to strengthen the gel, since physical hydrogels are known to be mechanically weaker compared to chemical hydrogels [81]. The obtained hydrogel shows excellent biocompatibility in vitro, and were able to host hydroxyapatite nanoparticles on its surface to enhance bone regeneration, hinting the possibility for similarly constructed hydrogel to be used as a hydrogel matrix for CP-based composite hydrogel scaffold.

### 2.3. Hydrophilicity

Natural tissues and ECM are hydrophilic in nature, and therefore are more likely to attach to a hydrophilic surface. However, commonly utilized CPs are inherently hydrophobic in nature, thus leading to unfavorable cell-substrate interaction. This may negatively affect the process of cell adhesion and attachment of other necessary biological molecules to the scaffold’s surface, subsequently causing major issues in biocompatibility and may cause inflammation [18]. Furthermore, the immune system in the human body detects hydrophobic substances as foreign objects due to their significantly different properties from natural hydrophilic tissues, thus the introduction of hydrophobic pure CPs may trigger an unwanted foreign body response from the immune system [82]. This has become one of the reasons why blending CPs with biocompatible and hydrophilic non-conductive polymer has become the norm for electroactive scaffolds (aside from mechanical properties), with hydrophilic polymers such as chitosan [83] and alginate [84] being some of the attractive choices. As mentioned, this part is closely linked with the previous sub-section as both are discussing about the ability for the scaffold to facilitate cell attachment and growth. That being said, the following section will focus more on the surface properties of the scaffold to achieve favorable biocompatibility and bioactivity.

To promote hydrophilicity, there are two general approaches that can be used. The first approach is to deposit hydrophilic functional groups to the CP, which can be done either by grafting and creating a copolymer [85] or surface coating which can be achieved by substrate growing or by surface treatments such as plasma treatment [86]. Dopamine (DA)—which contains various functional groups including amine, imine, and catechol—can be used as universal anchor for surface modification, as was demonstrated by Tan et al. in their study for the development of DA-modified PANI [85]. The DA-modified PANI are shown to be dispersable in water due to the hydrophilic catechol group found in DA, whereas the hydrophobic unmodified PANI was completely destabilized and formed precipitate. Biocompatibility is greatly enhanced relative to pure PANI as a result of increased hydrophilicity, demonstrated by the high HeLa cell viability of 0.88 compared to 0.75 of pure PANI in the same concentration. Due to the non-conductive nature of DA, creation of DA-modified PANI results in compromised conductivity with an increased ratio of DA, although this can be resolved by adjusting the ratio between DA and PANI to the necessary level. Even though hydrophilicity is necessary to some extent in order to prevent inflammatory responses, some studies seem to suggest that excessive hydrophilicity may lead to hindered protein and cell adhesion, in a way so that there is an optimum range of how hydrophilic a substrate should be to prevent macrophage from attaching, while at the same time allowing specific protein pertinent to cell adhesion to be adsorbed [87,88]. For example, doping of PPy with hyaluronic acid (HA) is known to increase its hydrophilicity, and the hydrophilicity will increase with higher molecular weight (Mw) of HA (40° water contact angle at 35 × 10^3^ Da of HA, down to 19.4° at 30 × 10^5^ Da of HA) [88]. Even though hydrophilicity is increased, in vitro cell viability of NIH3T3 fibroblast and SH-SY5Y neuroblastoma were severely reduced in samples with higher Mw of HA, possibly because the specific protein that facilitates the cell adhesion cannot be efficiently adsorbed by the overly hydrophilic surface.

**Figure 4:**
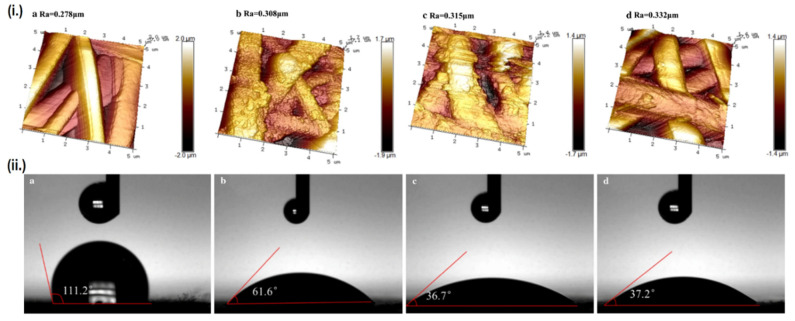
Relationship between surface roughness and hydrophilicity. (**i.**) AFM images and surface roughness value (Ra) of the scaffolds, and (**ii.**) Contact angle of each scaffold. Each alphabet corresponds to different samples, namely: (**a**) PLA, (**b**) PANI/PLA-HCl, (**c**) PANI/PLA-H_2_SO_4_, and (**d**) PANI/PLA-HClO_4_. Adapted with permission from [92]. Copyright (2021) Springer.

The other approach is by modifying the scaffold’s surface roughness, as higher surface roughness is known to promote hydrophilicity [89]. The creation of a CP-polymer composite may enhance the scaffold’s hydrophilicity even though the CPs themselves are hydrophobic, as the dispersed CPs particle will increase the scaffold’s surface roughness, although the effect is often minor due to the counteraction by CP’s innate hydrophobicity [90]. Another means of enhancing surface roughness is by using post-fabrication surface finishing, such as mechanical finishing, acid etching, or the previously mentioned plasma treatment. Interestingly, these two approaches can be simultaneously employed to impart greater effect on hydrophilicity. Aside from converting CPs from its non-conductive to conductive form, doping of acids can also be used to increase the scaffold’s surface roughness as a result of etching [91]. Liu et al. doped various inorganic acids (HCl, H_2_SO_4_ and HClO_4_) into PANI/PLA nanofibers to alter their surface roughness, while plasma treatment was also used in conjunction to imbue oxygen-containing groups (OH and COOH) onto the surface of PLA [92]. Ultimately, the combination of these two factors managed to significantly reduce the contact angle of PLA from 111.2° down to 37.2° when doped with perchloric acid, subsequently enhancing the biocompatibility as well. The perchloric acid-doped PANI/PLA nanofibers displays the highest cell viability and degradation rate, although the mechanical properties were severely compromised due to extreme surface roughness. On the other hand, samples doped with HCl and H_2_SO_4_ which presents less extreme surface roughness, displays much better mechanical properties, along with comparable biocompatibility and hydrophilicity as the HClO_4_ doped sample (Figure 4). It should be noted that not all acid doping will yield positive result, as another study by the same group reported decreased cellular attachment and proliferation when PANI is doped by PAMPS, possibly caused by a dramatic decrease in total surface energy, creating a huge gap between the total surface energy of the scaffold’s and the cell’s [93].

### 2.4. Biodegradability

**Figure 5:**
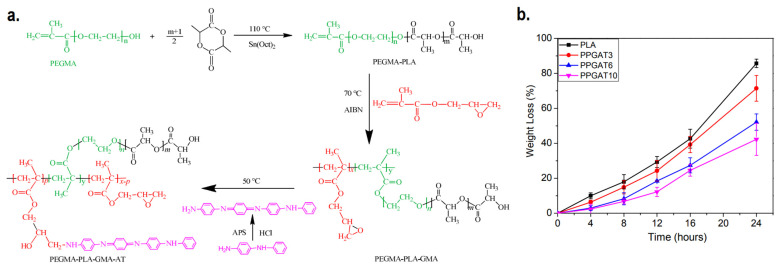
(**a**) Synthesis route of PEGMA-PLA-GMA-AT biodegradable conductive polymer. (**b**) Biodegradability of the copolymer with various ratio of PLA:aniline tetramer. Adapted with permission from [94]. Copyright (2016) Royal Society of Chemistry.

Biodegradable scaffolds which have the ability to undergo controlled degradation when implanted in vivo are desirable, as it makes the process of post-recovery implant detachment unnecessary. CPs homopolymers are not inherently biodegradable, and biodegradability itself is not something that is commonly expected in the current generation of CP-based electroactive scaffolds. Even though blending CPs with biodegradable polymers (e.g., chitosan, gelatin, alginate, etc.) will result in mostly biodegradable scaffolds, the CPs are expected to remain inside the body after the matrix has been degraded, potentially causing harmful effects afterwards due to its potential cytotoxicity [26]. For the development of biodegradable and intrinsically conductive polymers, a new class of materials must be developed, branching from the conventional CPs homopolymers. One promising example is the development of degradable electroactive polymers by combining oligomers of CPs along with biodegradable polymers, creating copolymers. Li et al. synthesized an inherently electroactive biodegradable polymer based on polylactide and aniline tetramer as a base material for electroactive scaffold [94]. The synthesis first began by adding poly(ethylene glycol) methacrylate (PEGMA) as initiator to PLA synthesis by ring-opening polymerization. The resulting PEGMA-PLA copolymer is then further copolymerized with glycidyl methacrylate (GMA) via free radical polymerization. This allows the aniline tetramer to be introduced to the copolymer to imbue electroactivity to the biodegradable copolymer, as a result of the interaction between the amine group of aniline tetramer and epoxy group from GMA. The synthesized film demonstrated up to 71% of degradation in NaOH solution within 24 h, although the increase in aniline tetramer percentage will decrease its degradation capability (Figure 5). Later, the same group utilized similar concept to create tetraaniline-b-PCL-b-tetraaniline block copolymer, and demonstrated its printability in additive manufacturing to fabricate electroactive biodegradable cartilage scaffold [95]. A similar approach can be used to fabricate biodegradable electroactive hydrogel. Tetraaniline-graft-oxidized alginate (OA) nanoparticles alongside OA can be used as crosslinkers to gelatin hydrogel, where the conductive nanoparticles would reinforce the hydrogel while simultaneously imparting electroconductivity, and the gels were able to mostly degrade (~65%) after 5 weeks of in vitro incubation in phosphate-buffered saline (PBS) solution [96].

## 3. Specific Improvement Strategies for Specific Body Parts

**Figure 6:**
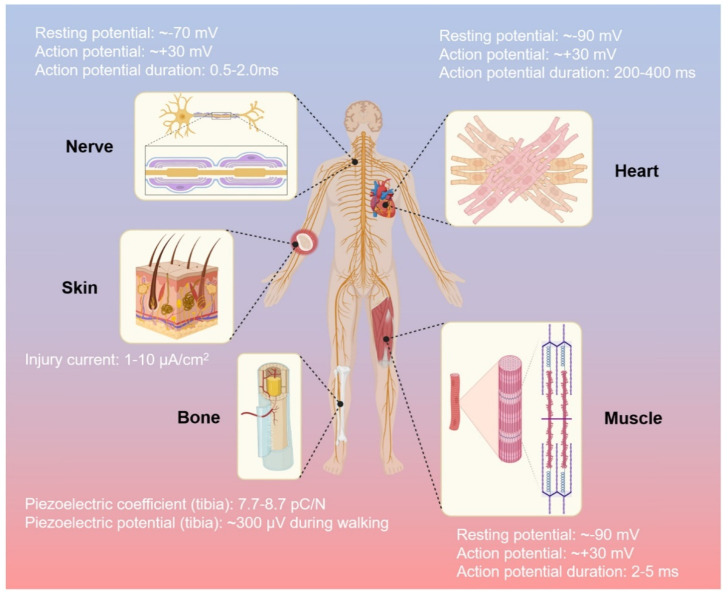
The human body and its electrical activity. Reproduced with permission from [97]. Copyright (2021) Elsevier.

Various types of tissues are regulated by electrical signals in the human body, such as activities of neural communication, heartbeat activities, bone regeneration, muscle contractions, and wound healing, respectively [97]. The important role of the electric biological field leads to tissues repair due to direct current and stable electric potential, causing cells to migrate to the wound site [98]. CPs allow fine-tuning of chemical, electrical, and physical properties to suit the needs of the tissue part in which they are used [99]. To obtain greater impact of electroactive scaffolds, their implementation should fulfill not only general characteristics of biomaterials (that were already described in Section 2), but also specific needs of each tissue that might vary with other tissue due to the difference of extracellular matrices and function. Various electrical activities of each tissue in the human body as the guideline of specific requirements for implementation of electroactive scaffold in each tissue are described in Figure 6 [97]. Beside electrical conductivity, certain mechanical properties that specific to each tissue should also be considered, such as high compression strength and modulus for bone tissue and typical elasticity for skin tissue. Thus, this section will focus on the improvement of (i) required electrical properties of CP-based electroactive scaffolds that are suitable with designated tissue and their electrical stimulation phenomena, and (ii) certain mechanical properties of CP-based electroactive scaffolds that correlated with the target tissues (summarized at the end of this section in Table 1).

### 3.1. Bone Tissue Engineering

#### 3.1.1. Conductivity of Bone Scaffold

Bone has conductivity values around 1.6–2.0 × 10^−3^ S/cm and 5.8–6.3 × 10^−4^ S/cm for cancellous bone and cortical bone, respectively [100]. One of the common strategies to mimics the electrical properties of bone tissue is incorporating conductive fillers such as CPs to improve the conductivity value of the scaffold [101]. For example, our previous study showed that the conductivity of the pure PCL scaffold (1.1 × 10^−11^ S/cm) could be enhanced significantly by the addition of 0.1% wt PANI (2.46 ± 0.85 × 10^−4^ S/cm), which is within the region observed in cancellous and cortical bone [44]. Conductivity of CP-based electroactive scaffold could be improved by increasing the CPs concentration in the scaffold. However, it should be noted that the higher amount of CPs concentration in scaffold could also enhance their toxicity in biological environment [102]. Thus, finding the optimum CPs concentration in the scaffold that provide a sufficient conductivity for bone tissue with less toxicity is crucial for the application of CPs-based electroactive scaffold on bone tissue engineering. The optimum PANI concentration of 3D printed PCL/PANI scaffold was 0.1% wt, because increasing PANI concentration on the scaffold by 1% wt and 2% wt led to elevating their cytotoxicity with only slight improvement of the scaffold’s conductivity [44]. It is noteworthy that they used melt blending method to prepare pre-mixed of PCL and PANI, which is a facile mixing method and suitable for scaffold manufacturing through 3D printed method without utilization of any toxic organic solvents.

The same tendency was also observed in electroactive scaffold based on other CPs, such as PPy as observed by Zarei et al. [53]. They prepared conductive polypyrrole/chitosan/collagen electrospun nanofiber scaffold with varied PPy concentration (0, 5, 10, 15, 20 and 25% wt) and crosslinked by glutaraldehyde vapor (denoted as PPCC, PPCC5, PPCC10, PPCC15, PPCC20 and PPCC25 respectively). They found that the conductivity of PPCC, PPCC5, PPCC10 and PPCC15 were 0.8, 1.2, 1.5 and 1.6 × 10^−3^ S/cm respectively. The scaffold conductivity enhanced as PPy concentration increased might be the result of more contact between the conductive polymer particles, both on the surface of the fiber and within it. Interestingly, all scaffolds showed low cell toxicity regardless of their PPy concentration due to the presence of bioactive ingredients such as chitosan and collagen that able to stimulate cell proliferation, although the maximum performance was owned by the scaffold with 10% wt of PPy [53].

Beside increasing CPs concentration, utilization of different CPs morphology might be an attractive strategy to improve electrical properties of electroactive scaffold [11]. An example of this approach is the application of tubular and spherical morphology of PPy in PLLA/PPy electroactive scaffold [103]. It is known that the conductivity of the tubular PPy (4.8 × 10^−1^ S/cm) is higher than the spherical PPy (0.6 × 10^−4^ S/cm). As the result, employing the tubular PPy in composite scaffold (~7.0 × 10^−4^ S/cm) gave a conductivity value that almost four times higher than the spherical PPy (~1.8 × 10^−4^ S/cm), while maintaining their low cytotoxicity with cell viability is higher than 80% [103]. The possible reason for this phenomenon is that the tubular PPy could intertwine and contact between adjacent tubular PPy easier than the spherical PPy, so that the tubular PPy could produce higher conductivity than the spherical PPy at lower concentrations.

The other strategy to increase the conductivity of the electroactive scaffold is by adding organic solvents to the CPs solution during the synthesis process. Ruzaidi et al. prepared chitosan–gelatin–agar-PEDOT:PSS electroactive scaffold and PEDOT:PSS were synthesized through the chemical oxidative polymerization method [104]. They found that adding 3.0 vol.% organic solvent dimethyl sulfoxide (DMSO) into PEDOT:PSS solution during their synthesis could significantly increase their conductivity from 1.71 ± 0.01 × 10^−5^ S/cm to 3.75 ± 0.06 × 10^−1^ S/cm [104]. Even though DMSO is considered as non-toxic solvent at a concentration below 10% (*v*/*v*), its utilization on biomedical application should be avoided as much as possible because DMSO could induce alterations in miRNA and epigenetic landscape in the 3D maturing cardiac model at 0.1% DMSO [105] and induce retinal apoptosis at DMSO concentration above 1% [106].

**Figure 7:**
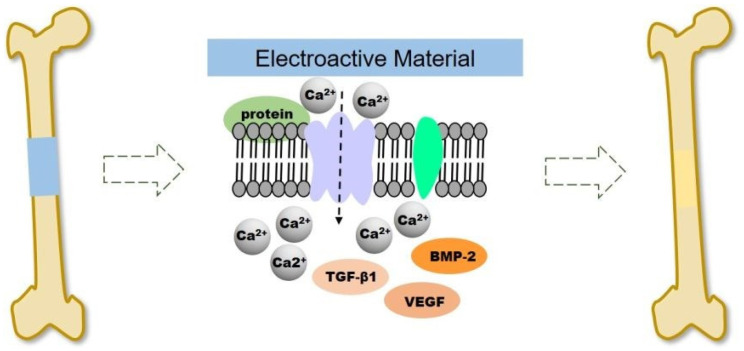
Schematic illustration of electroactive scaffold to induce bone regeneration. Reproduced with permission from [113]. Copyright (2020) Royal Society of Chemistry.

To optimize the utilization of the electrical properties of CPs at the injury site, improvements to the performance of the scaffold can be enhanced through exogenous electrical stimulation. The methods of providing electrical stimulation used in clinical practice are direct current and capacitive coupling [107,108]. Electrical stimulation (ES) can significantly promote the proliferation and differentiation of osteoblasts at the cellular and tissue level in a small amount of currents between 5 and 100 μA [108,109]. A representative example was carried out by He et al. by using a continuous micro constant current electrical stimulation signal (10 µA) on the surface of different substrates (PPy NWs and PPy/PDA NWs) via an electrochemical workstation for 12 h after cells inoculation and for 2 h per day for 14 days [110]. It was noticeable that continuous ES increase the adhesion and proliferation of MC3T3-E1 cell in PPy/PDA NWs, significantly. Also, the result showed ES addition could accelerate the cells entering the mature stage of osteogenic differentiation of osteogenesis differentiation. Electrical signals regulate cell proliferation and differentiation by controlling ion channels and altering the structure of the cytoskeleton in bone tissue [111]. As illustrated in Figure 7, ES causes the voltage gate Ca^2+^ channels on the cell membrane to open, allowing Ca^2+^ to enter the cells, resulting in intracellular Ca^2+^ concentration increased, and activates the expression of various growth factors such as transcriptional transforming growth factor (TGF). -β) and bone morphogenetic protein (BMP) [112,113].

In capacitive coupling, bone stimulation has been shown to be efficient by using a potential of 1 to 10 V at frequencies between 20 and 200 kHz, which creates an electric field of 1 to 100 mV/cm [108]. In another way, Maharjan et al. applied modified DC pulse stimulation (200 mV/cm) at a frequency of 100 Hz at 50% duty cycle for 1 h every day on PCL/PPy scaffold, which improved alkaline phosphatase (ALP) activity and alizarin red S (ARS) staining that significant for osteogenic differentiation [114]. Applied a potential at bone tissue, will increase electrical potential at the fracture site. Hereinafter, electroactive materials can increase local ES and assist in rebuilding the electrophysiological microenvironment to promote bone regeneration. Cells are recruited from the surrounding tissue to or into the electroactive material due to the galvanotaxis effect [115]. The mechanism of this effect is that protein adsorption will increase due to the ability to attract ions possessed by the surface charge of the electroactive material, which can bind to cells through ionic or charge interactions. As a result, cell migration, adhesion, proliferation, and osteogenic differentiation via activation of growth factor expression (eg., BMP, TGF-β) with locally generated ES will be enhanced by electroactive material [113,116].

#### 3.1.2. Mechanical Properties of Bone Scaffold

The common issue is CPs are very brittle, as we mentioned in the previous section. This property is opposite with the requirement of bone scaffold that needs strength and ductility to avoid brittle fracture. CPs like PPy, PANI, and PEDOT:PSS have Young’s modulus value of 180 MPa, 1.3 GPa, and 2.7 GPa, respectively which is very low compared to Young’s modulus of bone, especially cortical bone [117,118].

To overcome this problem, the mechanical properties of CPs can be optimized by doping or combining the CPs with metals, ceramics, or other polymers that have higher mechanical properties than CPs. For instead, fabrication electroactive bone scaffold mesoporous silica PPy-based through solvent casting method improved the young’s modulus (0.11 GPa) and compressive strength (7 MPa) of scaffold to have similar properties with cancellous bone [119]. Created a mesoporous silica PPy-based scaffold made the scaffold more porous and less dense, which decreased the young’s modulus (0.125 GPa) and compressive strength (8 MPa) of pure mesoporous silica but still within the allowable range.

An interesting approach to improve the poor mechanical properties of CPs-based scaffold was conducted by Ghorbani et.al. They decorated the electroactive scaffold with PU-PANI/PVA/PDA through the electrospinning method [120]. It was known that polyurethane (PU) has high mechanical properties that will resolve the brittle issue of PANI. The result showed that scaffold has 34.06 ± 1.16 MPa tensile strength showed higher similarity to the bone and 24.75 ± 2.32 MPa young’s modulus, which is in the range of cancellous bone. Another polymer that is used as an alternative to obtain good mechanical properties from CPs-based scaffold is polyethersulfone (PES) which is a biocompatible material. Pournaqi et al. used the electrospinning method to fabricate the PES/PANI scaffold to mimic the physiochemical structure of native bone tissue ECM [121]. PES/PANI nanofibers showed tensile strength of 1.85 ± 0.365 MPa, which was in the cancellous bone region.

Apart from that, incorporating CPs with metals would be the best strategy to obtain the higher mechanical properties due to high mechanical properties of metals. This strategy has been carried out by Jie et al. who fabricated bone scaffold using rGO/PPy through electrostatic LBL assembly strategy, followed by an electrochemical deposition process results in better mechanical properties and can be processed into the desired configuration [122]. The 3D rGO/PPY scaffold has a hardness value (92.27 ± 4.03 MPa) and Young’s modulus (185.94 ± 10.76 MPa) almost twice as high as the hardness value (48.59 ± 4.96 MPa) and Young’s modulus (91.0 ± 4.19 MPa) of 3D rGO met the requirements of clinical surgery for trabecular defect repair [122].

### 3.2. Nerve Tissue Engineering

#### 3.2.1. Conductivity of Nerve Scaffold

The neural network within the human body plays a distinct and important role in all physiological processes, including cell recognition, sensory and motor functions. The application of electroactive scaffold is attractive and promising to further promote the growth and differentiation of neurons and the formation of neural networks. Wang et al. showed that scaffolds can be considered as suitable candidates for electrical stimulation of cells if they have a conductivity around 1.0 × 10^−3^ S/cm [123]. To meet the conductivity requirement of neural tissue, the CPs can be coating, doping, or blending to a scaffold as a strategy to improve electrical properties.

Incorporation of the conductive polymer PEDOT:PSS to produce a scaffold with the required conductivity was applied to a silk fibroin (SF) scaffold [124]. The SF scaffold was fabricated using the electrospun method and then submerged into PEDOT:PSS and DMSO-treated PEDOT:PSS solutions. It is known that the isoelectric point of PSS and SF is at pH 1.2–1.5 and pH 4–5, so that the optimal submerged process can be carried out in an acidic environment (positively charged polypeptide fiber) [125,126]. An acidic environment conditioning (~pH = 2) was carried out to ensure that the silk and PEDOT:PSS were at a high level of electrostatic attraction during the process. Measurement of the conductivity value showed that neat silk has a very low conductivity (~10^−6^ S/cm), while the conductivity value is higher and tends to increase with the addition of PEDOT:PSS concentration (range ×10^−5^ to 10^−1^ S/cm). Moreover, the conductivity values of the DMSO-treated scaffold were much higher than that of the other samples (range ×10^−5^ to 4 S/cm), which tended to increase with the addition of PPy concentration (solvent concentration of PPy ranging from 0.5 to 13 mg/mL). Treatment with DMSO which is a polar solvent leads to optimization of the conductivity value of PEDOT:PSS. Polar solvents cause the anionic shell of PSS to be partially washed away, causing a shift in the PEDOT:PSS structure which decomposes from the coil conformation (benzoid) to linear extended coil conformation (quinoid), so that the undissolved PEDOT:PSS results in efficiency in changes in orbital overlap and packing between chain. Then a better pathway for charge transport is available which leads to an increase in conductivity [127]. DMSO is an example of a solvent treatment that is often used, but other types of solvent treatment commonly used are methanol, dimethylformamide, tetrahydrofuran, or ethylene glycol. Improvements made to SF/PEDOT:PSS and SF/DMSO-treated PEDOT:PSS scaffolds did not show major cytotoxic effects and were able to modulate growth and differentiation of NG108−15 cells in vitro better than pure SF scaffolds. SF/DMSO-treated PEDOT:PSS scaffold with 3 mg/mL PEDOT:PSS showed the most optimal results compared to other concentration variations in testing metabolic activity, cell proliferation, and neuron differentiation.

Improvement of the conductivity of the scaffold with an increase in the concentration of CPs when blended with other materials was also observed in the PCL/PPy scaffold. Sanjairaj et al. varied the concentration of PPy (0.5%, 1%, and 2% *v*/*v*) when fabricating a porous 3D scaffold from block copolymer PPy and Polycaprolactone (PPy-b-PCL) which can be decomposed through a novel electrohydrodynamic 3D jet printing process [128]. Significant improvement occurred due to the addition of PPy to the scaffold, where the measured conductivity of pure PCL was 0.09 S/cm, much lower than PCL/PPy 0.5% (28 mS/cm), 1% PCL/PPy (1.02 mS/cm) and PCL/PPy 2% (1.15 mS/cm). Very low conductivity values in PCL scaffolds are undesirable in neural network scaffold applications, as conductivity is a desirable property of ideal neural guiding channels (NGC) such as peripheral nerve regeneration. As was the case with the previous use of PEDOT:PSS, an increase in the concentration of CPs which results in higher conductivity is not always in line with optimal biological activity. The 1% PCL/PPy scaffold showed optimal results in biological activity characterized by strengthening the differentiation and maturation of hESC-20 NCSCs to peripheral neurons.

Eftekhari et al. did the fabrication of the scaffold using a different conductive polymer of PANI [129]. PANI was blended with CS to produce a conductive scaffold in the form of cell-imprinted hydrogel on the differentiation of mouse adipose-derived stem cells (rADSCs) into neuron-like cells. It is known that chitosan is a non-conductive material with a conductivity value of 7.5 × 10^−8^ S/cm so that blending with PANI will improve the conductivity value of the scaffold which is also in line with increasing PANI concentration. PANI with high -conjugated system, affected the electrical conductivity of the blend materials, strongly [130]. Increasing the amount of PANI from 0% to 2.5 wt% increased the conductivity value of the scaffold from 7.5 × 10^−8^ to 10^−4^ S/cm, which is sufficient for applications in neural tissue engineering [131]. However, PANI at certain concentrations can be toxic to cells, so PANI must be used at optimal concentrations to be compatible with cells. The results of the MTT test showed that the scaffold at all concentrations of PANI had low toxicity to ADSCs.

**Figure 8:**
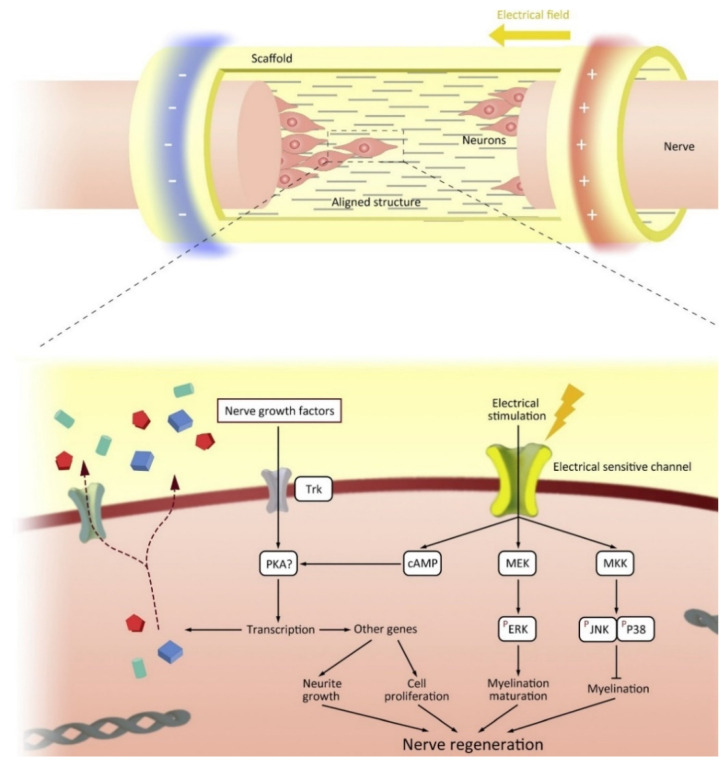
The mechanism of how electroconductive materials trigger the nerve regeneration through intracellular signalling. Reproduced with permission from [136]. Copyright (2020) Elsevier.

The conductive properties of conductive scaffolds can be utilized through electrical stimulation. When an injury occurs, neural cells will migrate to the site of damage to regenerate the damaged area. However, the microenvironment does not support the neural cells due to the damage that occurs resulting in the loss of cells and ECM. Electroactive scaffold will guide neural cells to the site of damage by mimicking the ECM required for the path to the site of damage [132]. Interestingly, neurons transmit information via synaptic and electrical junctions whereby neuronal signaling occurs both within and between nerve cells via the entry and exit of ions (sodium (Na^+^), potassium (K^+^), and calcium (Ca^2+^) along the axon [133]. Intercellular electrical signaling pathways are formed by differentiation of neural stem cells to form axons and dendrites, and proliferation of Schwann cells (SCs) aids neuronal development by growing myelin sheaths [134]. Synaptic processes are a combination of electrical and chemical events, the growth and differentiation of neurons can be regulated as a result of electrical stimulation [135]. The interaction between the nerve cell and the electroactive material is shown in Figure 8. Thus, the application of electroactive scaffold and electrical stimulation can increase the growth and differentiation of neurons and the formation of neural networks [132].

Zhao et al. applied ES to a PPy/SF conductive composite scaffold fabricated by 3D bioprinting and electrospinning, then compared it with a conductive scaffold not applied to ES to observe various cell behaviors under the influence of ES [136]. ES is done by providing a direct current of 100 mV/mm at different times. Cell viability observed in Schwann cells after 8 h of ES administration increased apoptotic cells and necrotic cells significantly and showed better cell proliferation than scaffold without ES. In the absence of ES, the PPy, SF, PPy/SF and blank groups showed SCs extension lengths of 28 ± 5 μm, 56 ± 3 μm, 65 ± 4 μm, and 22 ± 2 μm after 6 h. The long existence of SCs belongs to the PPy/SF scaffold. In addition, an increase of 140–170% occurs when ES is applied. These results demonstrate the important role of PPy in admixture with SF as a conductive scaffold and the impact exerted by ES application on the scaffold. The application of ES to the electroactive PPY/SF scaffold aligned the SC arrangement and its proliferation on the scaffold (marked by the finding of S100 and EdU positive cells). The administered ES also facilitates SC migration so as to provide contact guiding cues for anchorage and elongation and regeneration of axons [137]. The application of SF/PPy and ES scaffolds was able to increase neurotrophic factor, BDNF, and NT-4/5, as well as activate protein kinases of the MAPK signaling pathway. These substances play a role in accelerating neuronal regeneration and controlling important cytological activity in response to stimulation and cell types [138].

Significant differences in cell length due to ES administration were also observed in the PANI/ITO scaffold [139]. On the surface of the PANI/ITO scaffold without ES, the neurite length of 15.57 ± 5.65 μm was quantified. After ES administration for 4 h with 100 A amplitude, 0.8 ms pulse width and 1s with a repeat interval stimulating biphasic rectangular current pulse gave optimal results with an increase to 35.87 ± 7.74 m on the PANI/ITO scaffold [140]. An increase in the number of PC 12 cells also occurred after applying ES to the scaffold. Differences in the optimization of scaffold properties with conductive polymers were also observed in electrical stimulation for PANI/ITO and ITO (without PANI) surfaces. The increase in DMEM protein adsorption of about 54% occurred in the PANI/ITO scaffold compared to the ITO scaffold under ES for 3 h. The high protein absorption is associated with the electric field formed between the two sides of the scaffold surface which causes the protein interaction with the scaffold to be stronger than without ES. In addition, ES may change the conformation of the protein lead to more protein attachment to the surface of the scaffold which further enhances cellular adhesion (increases neurite extension).

#### 3.2.2. Mechanical Properties of Nerve Scaffold

The mechanical properties of the scaffolds used for neural tissue engineering should mimic the mechanical properties of the ECM to promote the neural differentiation of cells. Physical cues are an important factor in designing an artificial ECM to guide cells because according to the mechanical properties of stem cells, niches can regulate cell behaviour such as attachment, migration, and differentiation [129]. The use of conductive polymers in electroactive scaffolds can decrease and increase the mechanical properties of the scaffold. Therefore, it is necessary to determine the optimal composition of the conductive polymer in the electroactive scaffold to obtain an improvement in the mechanical properties. A recent example of this was demonstrated by Tavakoli et al. [84]. The group revealed that scaffolds containing concentrations higher than 2 mL PPy were brittle, so they broke into pieces during the drying process and in their surface evaluation with a four-point probe. These results indicate that higher PPy concentration causes brittleness of the scaffold [141,142]. As a result, this group uses a composite ratio of 2:10 for PPy:alginate which produces suitable and optimal mechanical properties [84]. Therefore, considering the optimal concentration of conductive polymer will be the first approach to achieve improvement in mechanical properties. The increase in mechanical properties must meet the target mechanical properties so that the increase and decrease in the value of mechanical properties such as Young’s modulus, compressive strength, tensile strength, etc. is not a problem, as long as the values are similar to or close to the target mechanical properties. For example native human peripheral nerve strength is ~6.5 MPa [128], spinal cord ~1.02–1.37 MPa [143], and the Young modulus of brain tissue is 7.11–9.21 KPa [45].

Other conductive polymers such as PEDOT also show lower mechanical properties (tensile strength) than PVA materials in PVA/PEDOT:PSS electroactive scaffolds. Babaie et al. added 0.1% by weight of PEDOT to the pure PVA scaffold which caused a decrease in tensile strength from 14.2 MPa to 7.2 MPa [144]. PEDOT is known as a brittle polymer with high young modulus and low mechanical strength due to its chemical structure and the presence of a thiophene ring [145]. However, the improvement of the mechanical properties of the scaffold by increasing the PEDOT content could be the result of increased crystallinity, decreased structural defects and decreased fiber diameter [50,146]. In fact, structural defects can form and spread more easily in thicker fibers than in thinner fibers [50]. Therefore, conductive polymers in electroactive scaffolds have a unique role in the improvement of mechanical properties in neural tissue application. Most importantly, the scaffold has the necessary mechanical properties, which allow it to maintain its bulk architectural morphology without collapsing before the tissue can regenerate.

Huang et al. also fabricated a PEDOT scaffold combined with chitosan in the form of a hydrogel through electrostatic interactions between partially deacetylated chitin (degree of acetylation = 80%, DA80%) [147]. In chitosan, a decrease in the value of DA causes a decrease in the value of Young’s modulus. Young’s modulus is an important property in providing a stable microenvironment for cell proliferation and tissue regeneration during recovery, so a high modulus will be advantageous for a long-term healing process. Huang et al. used 80% chitin with Young’s modulus of 4.1 MPa and (1–3%) PEDOT to fabricate ChT-1% PEDOT, ChT-2% PEDOT, and ChT-3% PEDOT scaffold. PEDOT mixing decreased Young’s modulus insignificantly due to an increase in sacrificial bonds (electrostatic interaction). Measurement of tensile strength increased with increasing PEDOT ratio, with values of 1.93, 2.14, and 2.49 MPa. Moreover, in in vitro and in vivo activity, the scaffolds exhibited cytocompatibility that promotes neuronal cell attachment and proliferation.

Blended conductive polymer with graphene can be an alternative solution to improve the mechanical properties of the scaffold. Chen et al. added carboxylic graphene oxide to polypyrrole/poly-l-lactic acid via electrochemical deposition method to obtain C-GO/PPy/PLLA-composite for neural network applications [148]. Measurements of the average tensile strength showed a value of ~32.7 MPa for C-GO/PPy/PLLA which was higher than ~21.1 MPa for PPy/PLLA. This increase indicates the polarity interaction between the imino group of PPy and the carboxyl group of C-GO. The tensile strength value of ~26.4 MPa of C-GO/PPy/PLLA was also significantly higher than that of PPy/PLLA. This value satisfies the need for application to the rabbit sciatic nerve and human peripheral nerve having ultimate tensile values of ~11.7 and ~15.87 MPa, respectively [149], whereas C-GO/PPy/PLLA can induce functional recovery from in vivo testing of SD rat sciatic nerve repair.

### 3.3. Skin Tissue Engineering

#### 3.3.1. Conductivity of Skin Scaffold

Skin wounds are one of the most common wounds caused by burns, diabetes, trauma, surgery, wound bed, and aging problems [150]. Scaffold applications can expand the ECM, presenting potential opportunities for cell attachment, proliferation, and migration, ultimately leading to the development of new skin tissues regeneration (e.g., keratinocytes and fibroblasts) [151,152]. The development of electroactive scaffolds in skin wound management has a beneficial effect on enhancing innate wound repair processes such as local inflammation, cell infiltration, and neovascularization [153]. Skin is a sensitive tissue to electrical signals and has a conductivity from 2.6 to 1 × 10^−7^ S/cm, depending on the component [154]. Numerous studies have been in order to improve electrical properties of skin scaffold to meet the requirement of skin conductivity.

On the other hand, the deposited CPs have formed a coating at the surface of the scaffold improved conductivity as constituted a relatively homogenous and continuous path. Silk fiber (SF) coated with PPy and PANI resulted in a significant improvement in the electrical properties of the scaffold. Measurement of conductivity of pristine silk fibroin fibers is 1 × 10^−11^ S/cm, the bulk conductivity of pure PPY is 1.3 ± 0.1 × 10^−5^ S/cm, and the pure PANI conductivity is 0.8 ± 0.1 × 10^−4^ S/cm [155]. In contrast, PPY/SF and PANI/SF-coated exhibited conductivities of 2.2 ± 0.1 × 10^−5^ S cm^−1^ and 1.6 ± 0.1 × 10−4 S cm^−1^, respectively. Coated fibers show higher compatibility properties than pristine SF making them more suitable for tissue engineering. Tests on HaCaT cells showed that the cells adhered to the PPY-coated SF surface and were more effective at PANI. In addition, good cytocompatibility was also shown by PPY/SF and PANI/SF coated [155]. Another coating using nanometer-scale PPy was applied via in situ chemical polymerization method on the surface of electrospun polycaprolactone-gelatin (PCL-Gel) nanofibers [156]. The conductivity of PPy-coated PCL-Gel scaffold nanofibers with various PCL-Gel concentrations ranged from 4.6 to 5.8 S/cm in a reaction time of 7 min in the in-situ chemical polymerization process. The increase in conductivity reached a maximum value (11.3 ± 2 S/cm) when the polymerization reaction time was increased to 55 min. Meanwhile, a two-fold increase in oxidant in the reaction led to a slight decrease in the conductivity value (4.81 ± 0.5 S/cm) compared to a two-fold increased in pyrrole (3.6 ± 0.07 S/cm) [156]. Low oxidant-to-monomer ratio could reduce the conductivity value of PPy [157]. Apart from that, the application of PPy coating on the PCL-Gel has improved the conductivity of the scaffold because the conductivity measurements of the uncoated PCL-Gel fibers showed insulating properties [156].

Crosslinking of conductive polymer (PPy) to poly(glycerol-amino acid) (PGA) was carried out by Zhou et al. to produce controlled electrical conductivity, skin adhesive behavior, and photothermo-chemo scaffold tumor therapy [158]. The cross-linking between branched poly(glycerol-amino acid), polypyrrole@polydopamine (PPy@PDA) nanoparticles and aldehyde F127 (PGFP scaffold) resulted in a multifunctional scaffold with higher conductivity properties. The PGFP scaffold showed an increase in the conductivity value due to the presence of PPy from (1.9 ± 0.009) × 10^−3^ S/cm (without PPy) to (6.7 ± 0.008) × 10^−3^ S/cm (incorporated PPy) [158]. Blending PANI with chitosan (CS) also gives satisfactory results in the application of wound healing to skin tissue. Blended PANI/CS is done through electrospinning method. CS is a non-conductive material, which when combined with PANI provides the conductivity values required for wound healing applications. The highest conductivity values were obtained by mixing CS with a higher concentration of PANI, as expected. In this study, Moutsatsou et al. used a 1:1 ratio (CS:PANI) as the highest PANI concentration, which gave conductivity values of ~2.75 × 10^−5^ S/cm and ~0.5 × 10^−5^ S/cm for before and after neutralization, respectively. Cytocompatibility evaluation on CS/PANI performed by human osteoblast cells showed that CS/PANI at all concentrations was not toxic, even supporting better osteoblast attachment than chitosan control [158].

**Figure 9:**
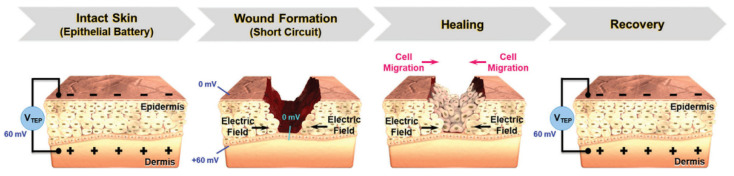
TEP and electric field at wound site before and after healing process. Reproduced with permission from [154]. Copyright (2021) John Wiley and Sons.

Electrical stimulation (ES) in wound care leads to the application of external electrical stimulation to tissues adjacent to, or directly within, the wound [159]. ES will accelerate the wound healing process due to it provides greater control over cellular differentiation and proliferation [159]. The mechanism that explains this function is that, in human skin, there is epithelial tissue that transports ions across the epidermis of the skin, forming a transepithelial potential (TEP) which is usually analogous to the ‘endogenous battery’ (Figure 9). When the wound penetrates the epithelial barrier, the TEP will drop because the epidermal battery is short-circuited. The internal electric current or TEP will decrease due to increased resistance during the skin healing process. ES operate by applying a small electrical field to the skin to mimic the TEP that occur during the granulation phase of wound healing [160]. ES that will promote wound regeneration and the conductive materials will support to distribute ES more effectively to the tissues in the wound [161].

**Figure 10:**
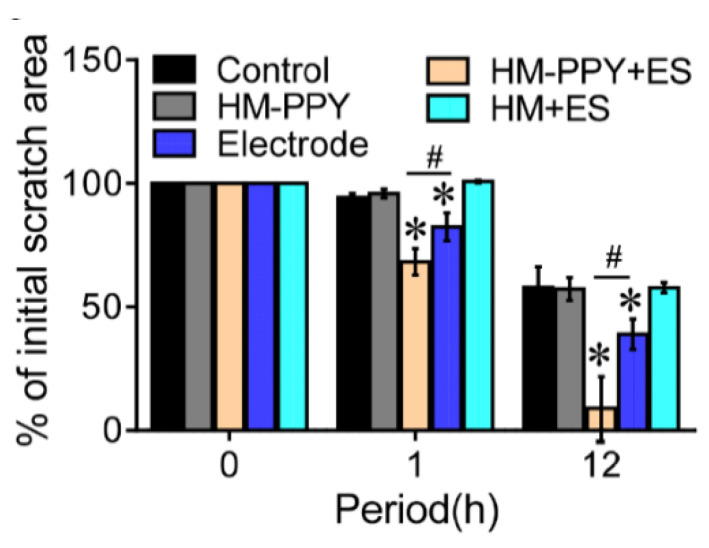
Quantification of % of initial scratch area for the different groups. * Significant difference with the control group (*p* = 0.05); # Significant difference between the designated groups (*p* < 0.05). Reproduced with permission from [37]. Copyright (2019) Elsevier.

Lu et al. have proven that applying ES to conductive materials accelerates the wound healing process through applied ES in the form of AC current with varying voltage and frequency to the fibroblast culture [37]. The optimization results show that the AC current with 5V, 40Hz for 1 h supports cellular metabolic activity significantly. ES was administered to HM-PPy for 1 h to 12 h and showed the remaining scratch area was reduced by 17% (1 h) and 77% (12 h) compared to the Electrode group. Another result showed that HM-PPy not treated with ES did not accelerate wound closure, indicated by a much slower scratch closure rate than HM-PPY+ES and confirmed the important role of ES in the wound healing process. The important role of PPy can be shown in Figure 10, where HM-PPY+ES showed a significant reduction in scratch area compared to HM+ES, where the application of ES requires sufficient conductivity [37]. The current intensity through PPY is estimated to be 10 times higher than HM based on its conductivity value. Therefore, the large electric current through the PPY component in the hydrogel could contribute to the initial closing process for the HM-PPY+ES group.

The electrical response activity of the scaffold was also investigated by Niu et al. on human skin fibroblasts through the PLLA/PEDOT scaffold through a two-step approach (melt spinning and melt spun) followed by an oxidative polymerization process [162]. ES was applied to the scaffold through a constant DC potential gradient of 40 mV/mm for 48 h. After that, the culture medium was changed and ES at 50 mV/mm for 6 h was applied to the cells. After the ES had ended, the cells were cultured for another 48 h and evaluated. The MTT assay showed a less significant increase in cellular activity, but still demonstrated the feasibility of using ES to mediate cells cultured on the scaffold. In addition, the scaffold did not indicate cytotoxicity and also able to maintain its electrical conductivity in culture media [162].

#### 3.3.2. Mechanical Properties of Skin Scaffold

The design of mechanical properties on skin scaffold engineering must be considered carefully because the development of skin substitutes is a functional performance that is closely related to mechanical strength. Cellular behaviours such as cell viability, cell-matrix interactions, cellular phenotype, differentiation, and focal adhesion size are highly dependent on the mechanical properties of the scaffold. In addition, the mechanical integrity of the skin tissue in vitro and in vivo to integration and remodelling of the ECM is also affected the scaffold [163,164]. Therefore, fabricating a scaffold with mechanical properties similar to natural ECM is essential to ensure no mechanical failures that limit cell growth and behaviour. On the fabrication method side, there is no such method that produces a functional scaffold with strength and rigidity that is very similar to that of genuine leather ECM, whereas tensile strength and Young’s modulus of skin tissue are 5–32 MPa and 25 kPa–140 MPa, respectively [165,166]. Therefore, other alternatives are used for the improvement of scaffold mechanics such as chemical cross-linking, mixing, and copolymerization [167].

Wang et al. fabricated bacterial hydrogel cellulose/polypyrrole/carbon nanotubes (rBC/PPy/CNT) by dissolving the polymerized BC/PPy in aqueous NaOH/urea solution, then the CNTs were impregnated, and physical and chemical crosslinking was performed [168]. CNT mixing is expected to improve the mechanical properties of the materials. In addition, the good properties of PPy and CNT can be used as reinforcement of hydrogel so that it can increase cellular activity with ES to improve the wound healing process. The mechanical properties test showed that the addition of PPy and CNT greatly affected the mechanical performance of the hydrogel, where the highest value of the compressive modulus was achieved (38.7 KPa). This value is almost three times higher than rBC (13.75 KPa). The stress at fracture and fracture strain was 17.79 KPa and 75.15% also higher than rBC (6.96 KPa; 72.5%). The addition of PPy also increased the compressive modulus from 13.75 KPa to 33.3 KPa. The test results showed an increase in the 3D rBC hydrogel network due to PPy and CNT, where there was a strong hydrogen bond between the OH group of BC nanofibers with CNT and PPy. This reduces the space between nanofibers and then increases the hardness of the hydrogel [169]. In addition, at the stress of 2.5 KPa which is much higher than rBC (1.6 KPa), rBC/PPy/CNT is able to withstand repeated loads (100 cycles) [168]. The compact structure of the rBC/PPy/CNT hydrogel can support a uniform response to continuously applied forces. Overall, the highest and stable mechanical strength can be obtained by the rBC/PPy/CNT hydrogels through this strategy. Improvements in mechanical properties by PPy and CNTs are also in line with in vitro biological evaluations showing that electroactive rBC/PPy/CNT hydrogels have good biocompatibility for NIH3T3 cell proliferation. In addition, cell proliferation in rBC/PPy/CNT hydrogels after application of ES was significantly increased compared to rBC hydrogels.

Another strategic approach was carried out by Razak et al. through the solvent-freeze extraction method where the conductive PANI was mixed to PLA [170]. The mechanical properties of PANI were improved by mixing it with PLA, wherein the increase in the PANI concentration on the PLA/PANI scaffold was directly proportional to the decrease in the tensile strength of the scaffold. The increase in PANI concentration on the PLA/PANI scaffold up to 4 wt% caused a decrease in the tensile strength value which was not too significant. Test results showed that PLA/0.5PANI has a tensile strength of 3.37 ± 2.2 MPa and PLA/4PANI has a tensile strength of 3. 08 ± 1.3 MPa, then increasing the concentration of PANI in PLA/5PANI reduces the tensile strength value a half to 1.58 ± 4.7 MPa. The decrease was due to PANI being brittle and the PANI chain conjugate acting as a non-reinforcing filler [171]. The PLA/5PANI samples showed a significant decrease in mechanical strength due to the larger PANI network. Hence, it is necessary to calculate the optimal number of PANI in tissue engineering applications in order to obtain proper mechanical properties. Optimization carried out by Razak et al. showed that the PLA/4PANI scaffold is a suitable scaffold based on its biological properties which is able to facilitate cell growth and good DC conductivity level, despite a significant reduction in its mechanical strength [170].

Chemical oxidation polymerization approach is one of the strategies used in scaffold fabrication. Massoumi et al. used this method to produce PEGs-b-(PPy)4 from PyPEGs macromonomers obtained from Steglich esterification of PEGs(OH)4 using pyrrole-2-carboxylic acid [172]. Solution of the electrospun-synthesized PEGs-b-(PPy)4 copolymer and PCL to produce nanofibrous scaffolds. This method produces a scaffold that has linear elastic properties before failure. Scaffolds were fabricated in two types based on differences in PEG molecular weight, where PEG (electrospun nanofiber PEG6000-b-(PPy)4/PCL) with higher molecular weight showed Young’s modulus (115 ± 4.1 MPa), tensile strength (9.2 ± 0.57 MPa), and higher elongation at break (46.6 ± 3.4 MPa) than PEG with lower molecular weight (electrospun nanofiber PEG2000-b-(PPy)4/PCL) with Young’s modulus value (108 ± 3.2 MPa), tensile strength (7.4 ± 0.46 MPa), and elongation at break (40.1 ± 2.7 MPa). The two fabricated electrospun nanofibers showed no significant toxicity and had excellent biocompatibility, as well as in vitro biodegradability, electroactivity, and proper conductivity in the presence of PPy [172,173].

### 3.4. Skeletal Muscle Tissue Engineering

#### 3.4.1. Conductivity of Skeletal Muscle Scaffold

Comprising about 45% of the total body mass in humans, skeletal muscle tissues are responsible for generating forces for various biological motoric functions. Muscles can be seen as electromechanical actuators, which converts electrical energy delivered from the nervous systems into mechanical energy. It is no surprise that electrical conductivity is a necessary factor to consider when attempting to mimic the functions and behaviors of muscle tissue. Skeletal muscles have a high capability of regenerating itself from minor injuries. Nevertheless, severe injuries resulting from major traumas, or medical causes such as myopathy or prolonged denervation, often results in irreversible loss of muscle functions [10]. Due to its ability to mimic the muscle tissues function as electromechanical actuators, CPs have gained attention in the development of electroactive muscle scaffolds. These actuators are noted to even exceed the performance of natural muscle tissues in terms of work density, making them desirable for replicating many muscle-like actions both inside and outside the body [174].

The mechanism in which CPs can act as actuators are due to the dimensional change produced as a result of insertion and de-insertion of electrochemical ions. When positive voltage is applied to the CP electrode, electrons will leave the CP, creating an imbalance in charge. This attracts the mobile anions found in the matrix to be inserted to the polymer to balance the charge, leading to expansion in the dimension of one electrode. In the case of antagonistic asymmetric architecture (i.e., two layers of CPs, each corresponds to a different electrode), the opposite process can occur in the counter-electrode, where the expulsion of ions will cause contraction of the electrode. Together, the expansion and contraction on the opposite side will cause the structure to bend, serving as the basic mechanism for electromechanical actuation based on the Faradaic principle. Skeletal muscle scaffolds can be divided into three categories based on its architecture: (1) monolith, (2) bilayer and (3) trilayer. A monolith structure consists of CP mixed with other polymers made into a singular structure, with CP-hydrogel composite being one such example [34,175]. Comparatively, this architecture has relatively low conductivity and unreliable mechanical stability, although the introduction of cations into the scaffold has been demonstrated to increase the conductivity of this approach [176]. Another approach is by creating a laminate structure, consisting of two or more layers of different materials bonded together into a single-layered architecture. These layers may constitute of two active CP components, or a CP component bonded with another passive layer of material [26]. Generally, scaffolds produced by this approach has significantly higher conductivity, as the electron mobility in the CP is not affected by the presence of other non-conductive polymer chains. A recent example of bilayer structure with passive layer was reported by Wang et al., who deposited PPy nano-/microstructured film on top of PET film [177]. Au was sputtered as coating to further reduce the resistance to allow lower driving voltage, and the bilayer shows bending angle of >480° when exposed to 4.5 V DC. As a continuation of bilayer structure, creation of trilayer structure may be beneficial in increasing the degree of actuation. In a trilayer structure, the middle passive layer is sandwiched between two CP layers, allowing for one side to contract while the other expand, resulting in improved performance over equivalent bilayer device. For instance, a trilayer architecture arranged as PPy-silk-PPy was fabricated, and was reported to demonstrate a larger magnitude of movement relative to its analogous PPy-silk bilayer devices with the same applied voltage [178].

Due to its heavy reliance on electrical impulses to carry out its actions, the conductivity requirement for skeletal muscle tissue is relatively demanding compared to many other tissues, sitting at ~1.25 × 10^−3^ S/cm [179]. As with other tissues, tuning the ratio of CPs to polymer matrix is often sufficient to achieve the intended conductivity value. Combination of PANI alongside PCL was reported to increase the scaffold’s conductivity value with increasing mass ratio of PANI to PCL [51]. At 3% wt PANI, the nanofiber sample managed to achieve conductivity value of 6.36 × 10^−2^ S/cm. The conductivity slowly diminishes over the duration of two weeks incubation in PBS. Due to the higher conductivity, the scaffold with 3% wt PANI managed to achieve the highest number of myotube present, longest myotube average length, as well as the highest maturation index. At the same time, it should be kept in mind that a higher concentration of PANI results in lower maximum elongation and higher stiffness of the scaffold. To compensate for the drop in conductivity for 3D scaffolds, as well as the gradual loss of conductivity over time, the doping strategy could be used to further enhance the nanofiber’s conductivity. Doping of glucose-gelatin nanofiber scaffold (CFS)/PPy with dodecylbenzenesulfonate (DBS) or trifluoromethanesulfonate (TF) was shown to be able to enhance the nanofiber scaffold’s conductivity, with TF-doped CFS/PPy showing higher conductivity (11.4 S/cm) compared to DBS-doped CFS/PPy (2.4 S/cm) [180].

**Figure 11:**
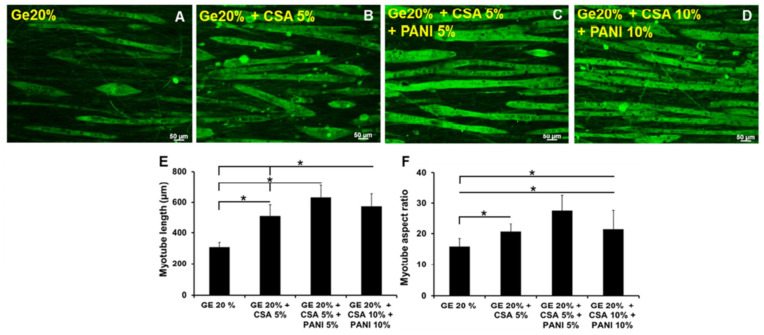
Effect of PANI addition to myotube formation and alignment. (**A**) Gelatin 20%, (**B**) Gelatin 20% + CSA 5%, (**C**) Gelatin 20% + CSA 5% + PANI 5%, (**D**) Gelatin 20% + CSA 5% + PANI 10%, (**E**) Myotube length quantification, and (**F**) Myotube aspect ratio quantification. * Significant difference between the groups (*p* < 0.001). Adapted with permission from [183]. Copyright (2017) American Chemical Society.

It should be noted that even without externally induced ES, the usage of electroactive muscle scaffold has an innate ability to support myogenesis, as the conductive nature of the scaffold provide electroactive cues to the newly attached myoblasts [181]. Nevertheless, ES can be applied to further boost the speed of recovery [182]. A study by Fortunato et al. managed to successfully incorporate ES to guide cell alignment and enhances myotubes differentiation [39]. Inkjet printing was used to fabricate layers of PEDOT:PSS on top of gelatin substrate, with higher layer counts (up to 50) providing better cell growth due to the lowered scaffold’s electrical resistance (and thus, lowered temperature as a side result of the applied voltage). Application of ES at 1 Hz managed to facilitate myotubes differentiation and alignment of 180° towards the applied voltage direction. In this case, the applied frequency also plays an important role in determining the ES effectiveness, as 1 Hz stimulation provided better result compared to 2 Hz stimulation and no ES. Different approach in the form of aligned scaffold structure could also serve as topographical cues to guide the elongation of skeletal muscle cell to one particular direction. Interestingly, this approach can be combined together with ES to increase the effectiveness of the overall scaffold in promoting tissue maturation [51,183]. The linear topography of PANI/gelatin nanofibers shown in the work of Ostrovidov et al. is shown to be favorable for the formation of parallel myotubes (Figure 11), and further introduction of ES to the conductive scaffold shows higher Ca^2+^ transient, which is necessary in skeletal muscle development and membrane fusion of myoblast during differentiation [183].

#### 3.4.2. Mechanical Properties of Skeletal Muscle Scaffold

With muscle being the type of tissue that undergoes a lot of repeated cycles of mechanical work, mechanical properties such as strain rate, tensile strength, elasticity modulus and cycle life becomes an important consideration. The elasticity modulus of muscle tissues range between 1–100 kPa, and substrates with similar modulus have been demonstrated to promote myogenesis [184]. A significant challenge in muscle tissue engineering is that these tissues experience high intrinsic stress from cellular contractions, thus demanding a robust yet elastic material. In the case of layered architectures, it is important to not only create an elastic electrode, but to create an elastic yet robust substrate as well.

Muscle tissue experiences a series of contraction and expansion. Therefore, the scaffolds must ideally possess high break elongation, as well as the endurance to withstand dynamic mechanical cycles without significant alteration in its properties. However, incorporating CPs into polymer matrix usually results in lower break elongation due to the brittleness of CPs. On the other hand, grafting and creating copolymer, as was demonstrated by Dong et al. in the fabrication of grafting aniline pentamer (AP) into poly(ethylene glycol)-co-poly(glycol sebacate) (PEGS) creating PEGS/AP copolymer, is an attractive strategy to create a conductive scaffold with high elongation at break [185]. With higher content of AP, not only conductivity was increased (1.74 × 10^−4^ S/cm at 9.3% wt AP), but elongation at break is increased (from 45.9% to 65.9%), and Young’s modulus is also enhanced (from 14.58 MPa to 23.46 MPa). This is likely attributed to the strong π-π interaction between the AP segments, and the covalent crosslinking network found in the structure also imbues the sample with good fatigue resistance property. Increasing content of AP also leads to better cell viability to a certain point due to increased conductivity to better match that of the natural myocardium, although overly high concentration ultimately leads to severely lowered biocompatibility, possibly due to the uncrosslinked leftover AP.

Citing comparable stiffness to muscle tissues and its naturally hydrated properties, hydrogels have emerged as an attractive candidate alongside CPs for muscle tissue engineering [186]. As discussed previously, hydrogels can be used to create a monolithic architecture [176], or to act as a passive layer in laminate architectures [60,182,187]. A recent study by Ting et al. shows that PPy-DBS grown on on poly(N-isopropylarcylamide) (PNIPAM) hydrogel exhibits more than two times the actuation of bare PPy-DBS [187]. Micro-patterned PEDOT polymerized on top of poly(ethylene glycol) (PEG) hydrogel was also reported to be highly biocompatible and supportive towards myogenic differentiation, as it was able to provide both electrical and topographical cues to the myoblasts [182]. The composite hydrogel exhibits a modulus of 45.84 kPa. Moreover, this scaffold was able to support electrical stimulation, further enhancing the myogenesis maturation. Utilization of tough hydrogel in muscle tissue engineering was demonstrated by Sasaki et al. who used a combination of chemical polymerization and electropolymerization of PEDOT and PU to form PEDOT/PU elastic electrode, which are then bonded onto DN hydrogel [58]. Without compromising electrical conductivity and biocompatibility, the utilization of double network structure managed to significantly improve the scaffold’s durability, which remains electrically stable after prolonged storage in aqueous media and repeated cycles of bending and stretching. With the PEDOT/PU electrode being similarly elastic as the gel substrate, the electrode structure and interfacial bonding between the electrode and the hydrogel substrate remained intact even after 100 bending cycles, whereas the PEDOT-only electrode shows rapid increase in resistance during the testing, suggesting that there might be a structural breakdown of the PEDOT chains due to its inherent brittleness.

### 3.5. Cardiac Muscle Tissue Engineering

#### 3.5.1. Conductivity of Cardiac Scaffold

Myocardium is an electrically conducting tissue, so the use of conductive materials has been made to mimic its intrinsic properties in repairing damaged tissue. However, the inhibition of electrical conductivity can occur during the process of fibrotic tissue formation or cardiomyocyte remodelling that impairs cardiac performance. Electroactive scaffolding is applied as a strategy to help repair and increase the electrical conductivity of the network so as to facilitate electrical connections between cells in the scaffold [67,188]. Therefore, a strategy is needed to obtain an optimal electrical conductivity value and in accordance with the cardiac tissue to support tissue repair.

A new strategy was devised to obtain a scaffold that mimics the properties of cardiac tissue. A new hybrid electro-conductive cardiac scaffold (CG-PPy) based on cardio gel (CG) derived from cardiac ECM and Ppy with different concentrations was fabricated (1%, 2.5%, 5%, and 10% *w*/*v*) [189]. In addition, PPy is doped with dilute iron (III) chloride (FeCl_3_). Doping on a conductive polymer will increase the value of its electrical conductivity, the doping process usually uses acid which changes the surface charge and is associated with electrostatic interactions between the scaffold and the cell [190]. This method yields high conductivity properties and is desirable for cardiac tissue engineering (CTE) applications. The conductivity value in the CG group was 0.007 S/cm, while in the CG-PPy group it was 0.023 S/cm. These results show that the mixed strategy of PPy with CG in the presence of FeCl_3_, as a dopant agent increased the conductivity of the scaffold almost 3.28 times that of the CG group. The CG-PPy electroactive scaffold with conductivity properties mimicking the structural conductivity of the ECM, is capable of transmitting signals throughout the volume scaffold. This impacted on the biological behaviour of the CG-PPy scaffolds compared to the CG scaffolds, where synchronization of NMCM cluster beating and increased expression of heart-specific genes (cTNT and Cx43) at 7 and 14 post culture days occurred in the CG-PPy scaffolds.

Doping PPy with acid (FeCl_3_) and blending with other materials is a widely used strategy to obtain the electrical conductivity required for CTE applications. Song et al. also fabricated scaffold with blended PPy into shell-PPy membranes by in situ polymerization to form conjugated PPy-chitosan shells using FeCl_3_ [191]. The shell-PPy scaffold has a conductivity of 6.2 × 10^−4^ S/cm which will benefit the electrical integration of the infarct zone and normal myocardium. In addition, electroactive scaffold with required conductivity is beneficial in enhancing cardiomyocyte (CM) function by increasing connexin 43 (CX43) expression. This aids in the regulation of cell-cell communication, enhances electrical connections, and promotes contractile behaviour in CTE.

The use of other types of dopant as a strategy in scaffold fabrication to improve the conductivity value of the scaffold was carried out by Almasi et al. [78]. Graphene oxide (GO) nanosheets were selected as dopants for the conductive polymer PANI in the fabrication of the polyacrylonitrile/polyaniline (PAN/PANI) scaffold by plasma treatment. GO has biocompatibility properties, high chemical activity, and the presence of an electron pair on the oxygen atom. In general, CSA is used as a dopant for PANI, but the release of CSA from PANi reduces cell proliferation as a function of the resulting acid medium, so the use of dopant is intended to avoid this problem. As a strategy to increase the conductivity value, a much higher and stable conductivity was generated as a function of the interaction through p-p accumulation and p-cation interactions [192]. The conductivity value for the PAN/PANI scaffold is 0.01 S/cm, while the conductivity for the PAN/PANI scaffold nanoparticles is 0.5 S/cm which is in the range required for CTE applications. The difference between scaffolds that have nano-size and does not show a difference in fiber diameter which results in the formation of fibers with a smaller diameter is related to an increase in the conductivity value [193]. In addition, in biological assays, an increase in cell-scaffold interactions and scaffold biocompatibility occurred in the presence of GO nanoparticles. In addition, the cardiac a-MHC antibody was expressed significantly with the presence of GO nanoparticles to bare type of scaffold.

In addition to the need for conductivity properties on the scaffold, the cardiomyogenic differentiation activity of peak cells is also influenced by electrical stimulation (ES) [194]. Electrical stimulation can effectively improve biological processes associated with tissue repair. Yang et al. compared the crosslinked PEDOT scaffold performance with Alginate (Alg) on Brown adipose-derived stem cells (BADSCs) under the influence of ES. The Alg/PEDOT scaffold had good conductivity for CTE applications (6 × 10^−2^ S/cm) applied electrical pulses (1.0 Hz, 1 V, and 2 ms pulses) to excite cells [195]. The effect of ES on BADSCs was investigated by measuring the expression of heart-specific proteins. In the case without ES, a low attachment capacity of the cells was observed in the pure alg scaffold, whereas the Alg/PEDOT scaffold was able to modulate the expression of heart-specific proteins. In the case of ES treatment, an effective increase occurred in BADSCs that differentiated into cardiomyocytes. Compared to the Alg/PEDOT scaffold without ES, the percentage of heart-specific proteins was clearly higher in the Alg/PEDOT scaffold with ES treatment. ES can activate the cardiomyogenic differentiation signaling pathway by increasing the production of intracellular reactive oxygen species [196], in addition to the conductive nature of the Alg/PEDOT scaffold increasing cell-to-cell communication.

#### 3.5.2. Mechanical Properties of Cardiac Scaffolds

Appropriate mechanical properties of conductive scaffolds are a requirement for CTE, so that appropriate strategies are needed in scaffolding design [197]. Abedi et al. made an improvement to the mechanical properties of the CS/PVA scaffold by adding PEDOT:PSS [50]. Chitosan has unique properties for tissue engineering applications but has limitations in its mechanical and electrical properties. Scaffold fabrication containing CS/PVA/PEDOT:PSS is carried out through the electrospinning method. The mechanical properties of the electrospun scaffold depend on several parameters such as the chemical composition of the polymer, the type of solution, the nature of the collector, etc. [198]. The results of the measurement of mechanical properties showed a higher Young’s modulus at a higher concentration of PEDOT:PSS and a lower diameter. Young’s modulus values for CS/PVA, CS/PVA/PEDOT:PSS (0.3), CS/PVA/PEDOT:PSS (0.6), and CS/PVA/PEDOT:PSS (1) respectively are 9 MPa, 12.5 MPa, 16 MPa, and 18 MPa, respectively. Furthermore, CS/PVA/PEDOT:PSS (0.6) and CS/PVA/PEDOT:PSS (1) have higher elasticity, this is due to fewer imperfections in the fibrillar structure and higher crystallinity. Higher fiber diameter results in more structural imperfections, which will increase fiber deformation at lower stress values occurring in CS/PVA (~9 MPa) and CS/PVA/PEDOT:PSS (0.3) (~12.5 MPa) samples. The CS/PVA/PEDOT:PSS (0.6) and CS/PVA/PEDOT:PSS (1) scaffolds have been shown to withstand up to 16.45 MPa and 18.78 MPa, respectively. In the CS/PVA and CS/PVA/PEDOT:PSS (0.3) samples, at the beginning of the deformation, the fibers began to break gradually indicating high fiber structure imperfections. Therefore the CS/PVA and CS/PVA/PEDOT:PSS (0.3) samples showed lower elasticity, toughness, and tensile strength. In addition, the hydrogen bonding between the OH groups in PVA and chitosan and SO_3_ groups in PSS from the conductive polymer dispersion also affects the mechanical properties of the scaffolds containing PEDOT:PSS [199,200].

Mawad et al. grew polyaniline (PANI) doped with phytic acid through polymerization on the surface of the chitosan film [201]. Measurement of the mechanical properties showed that the values of Young’s modulus, tensile strength, and elongation at break were 6.73 ± 1.14 MPa, 5.26 ± 2.25 MPa, and 79 ± 22%, respectively. The obtained mechanical properties are comparable to values reported for other PANI composites, such as poly(glycerol sebacate) containing 20 and 30% PANI [202]. Biological test results show that immobilizing dopants in conductive construction can provide the required mechanical properties.

**Table 1:** Non-exhaustive summary of CP-based scaffolds in various tissue engineering applications.

Tissue Type	Specific Requirements		Biomaterial (matrix/CPs)	Strategy	Optimum Result						Ref.
	Mechanical Properties (MPa)	Conductivity (S/cm)			Concentration (%)	Mechanical Properties (MPa)		Conductivity (S/cm)		Cell Viability/Proliferation	
						Pristine	Composite	Pristine	Composite		
Bone	Cortical [107]: Young’s modulus (*E*) = 6–20 × 10^3^ Compressive strength (*σ_e_*) = 106–224 Tensile strength (*σ*) = 51–151 Cancellous [107]: *E* = 50–500 *σ_e_* = 2–12 *σ* = 1.5–38	Cortical: 5.8–6.3 × 10^−4^ [100] Cancellous: 1.6–2.0 × 10^−3^ [100]	PCL/PANI	Incorporation PANI as a filler through melt blending method and suitable for scaffold manufacturing through 3D printing	0.1% of PANI	*E* = 5.33 *σ_e_* = 64.43	*E* = 6.45 *σ_e_* = 68.35	1.1 × 10 ^−11^	2.46 ± 0.85 × 10^−4^	Enhanced cell proliferation and cell viability from 75% to 88%	[44]
			Collagen/ chitosan/ PEO/PPy	Incorporation as PPy a filler through an electrospun nanofiber scaffold	10% of PPy	*E* = 6.04 *σ* = 15.1	*E* = 1.09 *σ* = 4.6	0.80 × 10^−3^	1.5 × 10^−3^	Enhanced cell proliferation and viability from 290% to 310%	[53]
			PLLA/PPy	Incorporation PPy as a filler with different morphology of PPy	15% of Tubular PPy	*σ* = ~120 *σ_e_* = ~18	*σ* = ~250 *σ_e_* = ~20	N/A	~7.0 × 10^−4^	Showed low cytotoxicity with cell viability around 80%	[103]
			PU-PANI/PVA/PDA	PVA incorporated into a PU-PANI/PDA scaffold through the electrospinning method	2% of PANI	*E* = 19.57 *σ* = 29.51	*E* = 24.75 *σ* = 34.06	0.9 × 10^−3^	0.7 × 10^−3^	Showed great ability in the biomineralization of hydroxyapatite-like layers (essential parameter in bone regeneration)	[120]
			Chitosan–gelatin–agar-PEDOT:PSS	Organic solvent (DMSO) added to the CPs solution during the synthesis process	Adding 3.0 vol.% DMSO	N/A	N/A	1.71 ± 0.01 × 10^−5^	3.75 × 10^−1^	DMSO is considered as non-toxic solvent at a concentration below 10% (*v*/*v*), but need to confirm by biological study	[104]
	Brain: *σ* = 0.5–1 × 10^−3^ [203] *E* = 7.11–9.21 × 10^−3^ [45] Spinal cord: *E* = ~1.02–1.37 [143] Peripheral nerve: σ = ~6.5 [128]	~3 × 10^−4^ to 6 × 10^−2^ [184]	SF/PEDOT:PSS	Organic solvent (DMSO) added to CPs and doped to SF	5.0 vol.% of DMSO in 3 mg/mL PEDOT:PSS	N/A	N/A	~10^−6^	~0.4	Enhanced metabolic activity, cell proliferation, and neuron differentiation	[124]
			SF/PPy	Incorporation as a coating with aligned diameter variation	Diameters and distances of coating are 180/700 (μm)	N/A	N/A	1 × 10^−11^	1.13 × 10^−3^	Showed good compatibility with L929 cells and may enhance Schwann cell adhesion, differentiation, and proliferation	[204]
			PCL/PPy	Fabricating porous 3D scaffold from block copolymer PPy and PCL through a novel electrohydrodynamic 3D jet printing method	1% of PPy	*E* = 204 *σ* = 18	*E* = 35 *σ* = 7	0.9 × 10^−7^	1.02 × 10^−3^	Promotes differentiation and maturation of hESC-20 NCSCs to peripheral neurons	[128]
			CS/PANI	PANI blended with CS to produce a conductive scaffold in the form of cell-imprinted hydrogel	N/A (0 to 2.5 wt% of PANI)	*E* = 7.0 × 10^−2^	*E* = 1.08 × 10^−1^	7.5 × 10^−8^	1.3 × 10^−4^	Showed biocompatibility and supportingrole of flat and cell-imprinted CS-PANI substrates for adhesion and growth of ADSCs	[129]
Skin	*E* = 0.0025–140 [165] *σ* = 5–32 [166]	1 × 10^−7^ to 2.6 × 10^−3^ [154]	SF/PPy and SF/PANI	CPs as coated material by in situ polymerization	N/A	N/A	N/A	1 × 10^−11^	2.2 × 10^−5^ (PPy) 1.6 × 10^−4^ (PANI)	Cells adhered better to the PANI-coated surface. Good cytocompatibility was also shown by PPY/SF and PANI/SF coated	[155]
			PGFP	Crosslinking between branched poly(glycerol-amino acid), polypyrrole/polydopamine (PPy/PDA) nanoparticles and aldehyde F127 (PGFP scaffolds)	5 wt % PPy/PDA	N/A	N/A	1.9 × 10^−3^	6.7 × 10^−3^	Showed outstanding rheological properties, controlled electrical conductivity and skin-adhesive behaviour	[158]
			rBC/PPy/CNT	Dissolving the polymerized BC/PPy in aqueous NaOH/urea solution, then the CNTs were impregnated (physical and chemical crosslinking)	N/A	*E* = 13.75 × 10^−3^ σ = 17.79 × 10^−3^	*E* = 38.7 × 10^−3^ σ = 6.96 × 10^−3^	3.47 × 10^−10^ (rBC)	1.67 × 10^−3^	Good biocompatibility for NIH3T3 cell proliferation	[168]
Muscle	*E* = 1–100 × 10^−3^ [184]	~1.25 × 10^−3^ [179]	PANI/PCL	Electrospinning to create aligned nanofibers of PANI/PCL	3 wt% PANI	*E* = 7.2 *σ* = 6.9	*E* = 55.2 *σ* = 10.5	N/A	6.36 × 10^−2^	76% cell viability vs. 63% of bare PCL (day 3). Aligned fiber increases number of myotube, myotube average length & maturation index relative to its randomly aligned counterpart	[51]
			CSA-PANI/gelatin	Doping of PANI/gelatin with CSA, fabricated with electrospinning	Gelatin 20% + CSA 5% + PANI 5%	*E* = 0.50	*E* = 0.51	9.1 × 10^−7^	4.2 × 10^−3^	34% myotubes matured compared to pristine sample (11%) at 4 days. Introduction of ES significantly increased the level of Ca^2+^ transient	[183]
			PEGS-AP	Copolymer creation by grafting AP onto the backbone of PEGS	9.3 wt% AP	*E* = 14.58 *σ* = 1.93 Break elongation = 45.9%	*E* = 23.46 *σ* = 3.91 Break elongation = 65.9%	N/A	1.74 × 10^−4^	2 × 10^8^ fluorescence intensity of live/dead C2C12 cells compared to 5 × 10^7^ of bare PEGS. Excessive AP loading beyond 9.3 wt% leads to significantly decreased biocompatibility	[185]
			PEG/PEDOT:PSS hydrogel	Micropatterned PEG hydrogel, followed by in situ PEDOT polymerization on top of the PEG substrate	N/A	*E* = 34.92	*E* = 45.84	N/A	2.49 × 10^−3^	Aligned micropattern is able to enhance myotube differentiation and aspect ratio by providing topographical cues alongside electrical cues from ES	[182]
Cardiac	*E* = 50–240 × 10^−3^ [205]	~1 × 10^−3^ [206]	CG-PPy	Doping PPy with FeCl_3_ and mixed with CG	N/A	N/A	N/A	0.007	0.023	Increased expression of heart-specific genes (cTNT and Cx43) at 7 and 14 post culture days occurred in the CG-PPy scaffolds	[189]
			PAN/PANI/GO	GO as dopants for the PANI in fabrication of PAN/PANI scaffold by plasma treatment	N/A	N/A	N/A	0.01	0.5	Increase cell-scaffold interactions, biocompatibility, and cardiac a-MHC antibody was expressed significantly with the presence of GO	[78]
			CS/PVA/PEDOT:PSS	Adding PEDOT:PSS to CS/PVA to fabricate scaffold through the electrospinning method	1% of PEDOT:PSS	*E* = 9	*E* = 18	6 × 10^−5^	7.63 × 10^−3^	Improves biocompatibility and cell viability	[50]
			Chitosan/PANI	Grew polyaniline (PANI) doped with phytic acid through polymerization on the surface of the chitosan film	N/A	N/A	*E* = 6.73 *σ* = 5.26 Break elongation = 79%	N/A	0.162	Early in vivo experiments indicates the scaffold did not induce proarrhythmogenic activity in the heart	[201]

## 4. Manufacturing Process

In order to realize an effective electroactive bio-scaffold, there are three factors that needs to be carefully considered: (1) the materials must be biocompatible, electrically conductive, sufficient in strength and similar in elasticity moduli with the replaced tissue; (2) the scaffold should be designed so that the morphology can be as precise as possible; and (3) its surface chemistry such as roughness, porosity and hydrophilicity must be in favorable conditions so that the implant can physiologically support recovery (i.e., by supporting cellular proliferation, nutrient transport, etc.). The second and third factors are directly tied to how the scaffold is designed and manufactured, whereas the first factor—although not directly related—also needs to be considered as materials selection can dictate whether or not a certain manufacturing process is feasible. For example, polymers such as PANI in itself is known to be difficult to process as it has limited solubility in common organic solvents, which makes it somewhat unsuitable to manufacture PANI-based scaffold using solvent casting. Therefore, methods that can rely on physical melting such as electrospinning [183] or additive manufacturing [44] can be chosen as an alternative instead.

Commonly used methods for the fabrication of CP-based scaffolds include solution casting [207], thermally-induced phase separation (TIPS) [64,208], gas foaming [209] and freeze-drying [210]. Certain methods have specific advantages, such as the simplicity of solution casting, or the ability to create highly porous structure (porosity over 95%) using TIPS [211]. However, as previously mentioned, these solvent-based methods require the polymer to be in the form of solutions, whereas many of the commonly used organic solvents (e.g., chloroform, acetone, dimethylformamide) have questionable biocompatibility in the human body [76,77,78]. In general, these methods offer little control to the morphology and geometries of the scaffold, which are some of the most crucial factors in ensuring the effectiveness and employability of the scaffolds.

### 4.1. Overview of Additive Manufacturing

Additive manufacturing—sometimes called rapid prototyping or 3D printing—is a manufacturing method that can build three dimensional structures based on a previously prepared 3D computer-aided design (CAD), in which the structure is assembled by adding the material layer-by-layer until all the layers have been printed, creating a faithful reconstruction of the 3D CAD model [212]. The greatest benefit of additive manufacturing compared to other conventional methods is the possibility of creating a reproducible and highly precise structures with complex geometries, thus allowing for greater personalization for each patient’s needs. Well-defined and interconnected porous structures can be reliably made in a 3D-printed structure, which allows for easier cellular attachments and integration to the host tissues, as well as facilitating nutrient and oxygen transport [213]. Due to the involvement of CAD blueprints before the actual scaffold fabrication and its high replication accuracy, the process of integrating numerical simulations to better predict the resulting scaffold’s mechanical properties becomes easier, with a recent study reporting good agreement (~83%) between the numerical simulation and the actual experimental results [214]. This allows for potentially reduced amount of experimental work required to tailor the scaffold’s properties. Moreover, additives such as drugs or electroactive fillers can be blended together with the polymer before printing, giving access to properties such as controllable drug release and electroactivity to a non-intrinsically conductive polymer [29,215]. Accordingly, additive manufacturing technologies have been demonstrated in the fabrication of various biomedical scaffolds that can accurately reflects the native in vivo environment [216].

Among all types of additive manufacturing processes, extrusion-based 3D printing, which includes fused deposition modeling (FDM, sometimes called fused filament fabrication or FFF) is regarded as the most commonly used method of additive manufacturing, both universally and specifically for electroactive biomedical scaffolds. In this method, thermoplastic polymer is loaded onto the feeder tank, which is then melted and extruded through a nozzle. During printing, the nozzle will move in *X* and *Y* axis according to the CAD design, creating a single layer before moving up in the *Z* axis to print the subsequent layer. Compared to other additive manufacturing methods, FDM is comparatively simple to operate, as the materials can be easily replaced, the process can be automated, and the operation itself is relatively low-cost.

In FDM, thermoplastic polymer is usually supplied in the form of filament. Commonly used materials for bio-scaffold production include PLA, ABS and PCL [217]. Although these polymers are not electrically conductive, the scaffold product can be made electroactive by mixing the CPs into the non-conductive polymer solution before printing. In our previous work, we blended PCL alongside PANI to fabricate a 3D printed electroactive bone scaffold [44]. PCL was added to the mix in order to overcome PANI’s post-synthesis brittleness, as well as to ensure that the overall scaffold have good biocompatibility, considering there are conflicting reports about PANI’s biocompatibility in the human body [64,65]. Mechanical mixing of PANI particles into PCL solution was used to avoid the use of solvents, and the process is suitable for melt extrusion utilized in FDM. The PANI were distributed evenly throughout the PCL matrix. Notably, the scaffolds were reported to possess regular and interconnected porous structure, as well as exhibiting suitable compressive strength and conductivity for electroactive bone tissue engineering application, with high cytocompatibility of up to 21 days.

**Figure 12:**
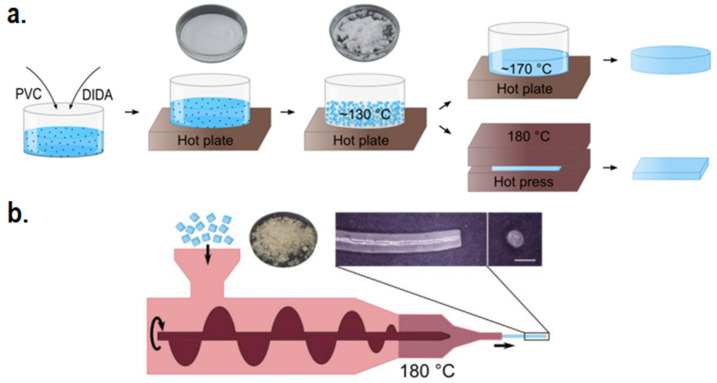
(**a**) Manufacturing route of PVC-DIDA electroactive thermoplastic gel. Heating at ~130 °C accelerates the speed in which the plasticizer is absorbed by the PVC, turning the plastisol into wet crumbly paste; (**b**) Extrusion scheme of PVC-DIDA gel. Adapted with permission from [218]. Copyright (2019) IOP Publishing.

Alternatively, electroactive thermoplastic gels can be used, as was demonstrated by Helps et al. for the purpose of developing 3D printable artificial muscles [218]. This gel material can be used similarly as thermoplastic filament, where the material can be heated and melted for further processing. This was done by mixing PVC with diisodecyl adipate (DIDA), which is a plasticizer with a flash point of 213 °C and boiling point of 349 °C. The normal solvent-based procedure uses dibutyl adipate (DBA) whose flash point is 113 °C, whereas PVC melts at around 170–180 °C, thus preventing the use of heat-based manufactures. The extrudability of this material was confirmed, and the group managed to fabricate an artificial muscle using additive manufacturing (Figure 12). Compared to solvent-based process, additive manufacturing process is significantly less time consuming (up to one week for solvent-based process, down to several minutes for additive manufacturing). Furthermore, additive manufacturing method that relies on physical blending and melting does not require solvents that are potentially toxic for use in human body.

### 4.2. Surface and Structure Improvement Strategies in Additive Manufacturing

**Figure 13:**
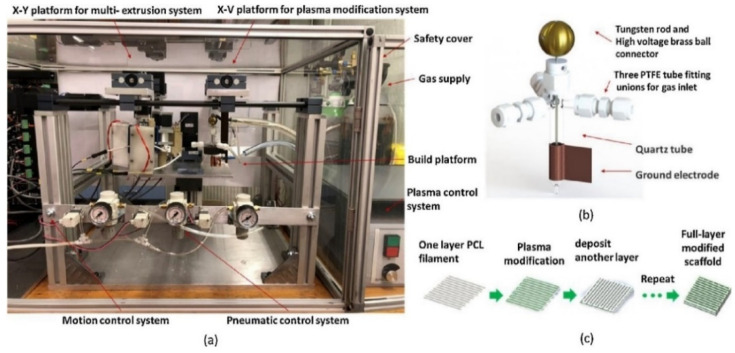
(**a**) Plasma-assisted bioextrusion system (PABS) 3D printer unit; (**b**) plasma modification unit; (**c**) sequence of operation for PABS 3D printing of bio-scaffold. Reproduced with permission from [223]. Copyright (2018) Elsevier.

An inherent problem with the FDM compared to many other types of manufacturing is the relatively poor surface finish of the product, which is unwanted, especially in the case of bio-scaffolds. Although this can somewhat be resolved by lowering the layer thickness, production time and cost will be severely affected. Another way to achieve better surface finish is by employing post-fabrication surface finishing, which can range from mechanical finishing (e.g., machining [219]) to chemical finishing (NaOH treatment [220], plasma etching [221,222]). In general, chemical finishing delivers a much more minute change compared to mechanical finishing, resulting in an ultra-smooth surface. Nevertheless, post-fabrication treatments require additional effort, and may theoretically lessen the consistency of the finished product. In response, Liu et al. introduced plasma-assisted bioextrusion system (PABS), which integrates plasma surface modification into the printing process (Figure 13) [223]. This system allows the printing of 3D structure alongside deposition of amine groups layer-by-layer in the same time by using nitrogen-based plasma modification, thus eliminating the need of post-fabrication finishing. Increase in the scaffold’s hydrophilicity was reported, signifying that ionizable groups were successfully introduced onto the surface as a result of plasma treatment. Surface roughness was also enhanced, which made the scaffold more suitable for supporting cell proliferation, as was confirmed during the in vitro testing. Similar one-step process of plasma treatment and printing was recently reported by Cámara-Torres et al., who uses hybrid platform to combine plasma jet with melt extrusion [86]. (3-aminopropyl)trimethoxysilane (APTMS) was used as a monomer for plasma polymerization on top of the printed fibers to deposit positively charged amine group. All plasma treated scaffolds reported higher hydrophilicity compared to untreated scaffold—even the control argon plasma which does not deposit any functional groups. Furthermore, the APTMS treated scaffold allowed cell attachment by electrostatic interaction, and when combined with the increase in hydrophilicity, results in enhanced bioactivity.

By modifying the processing parameter during 3D printing, the polymer’s microstructures can be altered, thus enabling the manufacturer to reliably improve the scaffold’s mechanical properties [224]. Tensile properties of PCL can be improved significantly by higher screw rotational speed, as there is a higher crystal volume fraction in the resulting scaffold due to shear-induced crystallization. On the other hand, lower processing temperature is noted to increase the elasticity modulus in a specific direction. A separate yet relevant study reveals that there is a strong directionality of the PCL crystals towards the direction of material flow when undergoing the extrusion-driven 3D printing process, which are less likely to re-align when the processing temperature is lower [225]. This anisotropic property is highly desirable for more accurately mimicking the nature of most human tissues. Looking at the scaffold’s macrostructures, pores and filament distance also have a huge impact on the mechanical properties, where higher porosity results in lower compressive modulus (46.0 MPa at 60.7% porosities, down to 6.0 MPa at 85.7% porosities). Furthermore, a well-controlled pore size at the range of 100 µm–300 µm are known to be the ideal pore size to facilitate cell adhesion and proliferation, as well as promoting vascularization [226].

Additive manufacturing can also be combined with another method of fabrication to achieve specific structural arrangements. Huang et al. reported the utilization of hybrid printing process, combining screw-assisted additive manufacturing technique with rotational electrospinning to fabricate dual-scale anisotropic PCL bone scaffold [227]. As extrusion-based additive manufacturing’s resolution is limited to microscale, electrospinning was employed to fabricate aligned fibers in the nanoscale, similar to that of extracellular matrix. As a result of the highly aligned nanofibers, higher cell seeding and proliferation can be seen in the dual-scale scaffold, with the cells observed being comparatively more elongated, showing higher anisotropic cytoskeletal organization than the scaffold produced with only 3D printing. In another demonstration, Munir et al. combined cryo-printing—a modified 3D printing method that prints directly onto a −40 °C surface—alongside electrospinning to recreate the complex multilayer architecture of human cartilage [228]. Cryo-printing of PCL/1,4-dioxane solution directly on top of a cold plate allows the printed solution to undergo phase separation and directional freezing, developing columnar pores similar to the parallel structures found in the deep zone of cartilage. Meanwhile, electrospun fibers are used to mimic the structures found in the superficial and middle zone of cartilage, with more aligned fibers on the superficial layer and randomly orientated fibers on the middle layer.

### 4.3. Cell-Laden Scaffold via Additive Manufacturing

Due to the high temperature involved in melting the thermoplastic, direct incorporation of cells and temperature-sensitive bioactive molecules currently still poses a significant challenge in conventional FDM or extrusion-based additive manufacturing [229]. If cells were to be seeded in a 3D printed scaffold, the commonly used procedure is to seed cells on pre-formed scaffold material. Recently, Spencer et al. developed a new approach in 3D bioprinting technology to construct pre-seeded cell-laden conductive hydrogel composite [230]. Spencer’s group utilizes photo-cross-linkable hydrogel electroconductive hydrogel consisting of gelatin methacryloyl (GelMA) mixed with PEDOT:PSS as the bio-ink for 3D bioprinting. Previously cultured cells were introduced into the GelMA/PEDOT:PSS hydrogel precursor solution, which were then detached and resuspended into the mix. Since the material is in the form of solution (liquid phase), the usage of high temperature to melt the material is unnecessary, and a temperature of 25 °C was used. As such, the loaded cells were unharmed during the whole manufacturing process. To solidify the printed structure, the scaffolds were exposed to photocuring light for 80 s to cross-link the hydrogel. While this approach is far more restrictive in terms of material selection as it requires the material to be photo-cross-linkable, this approach provides a facile way of combining the process of printing and cell seeding within one step, thus eliminating the need for post-seeding afterwards.

### 4.4. Stimuli-Responsive 3D Printed Scaffold (4D Printing)

As recent as 2013, 4D printing technologies have emerged as a form of advancement over its predecessor 3D printing technologies. Compared to the static object produced by 3D printing, 4D printing allows the printed structure to change in configuration with time in response to external stimuli (thus making “time” the other one extra dimension). Although the technology is very much still in its infancy, researchers have begun to implement 4D printing into various fields, including tissue engineering [231]. In order to make the printed structure responsive to stimuli, two approaches can be used: (1) by using smart materials that are inherently responsive to stimuli, or (2) by purposedly creating localized mismatch strain (commonly called eigenstrain) in the printed object.

**Figure 14:**
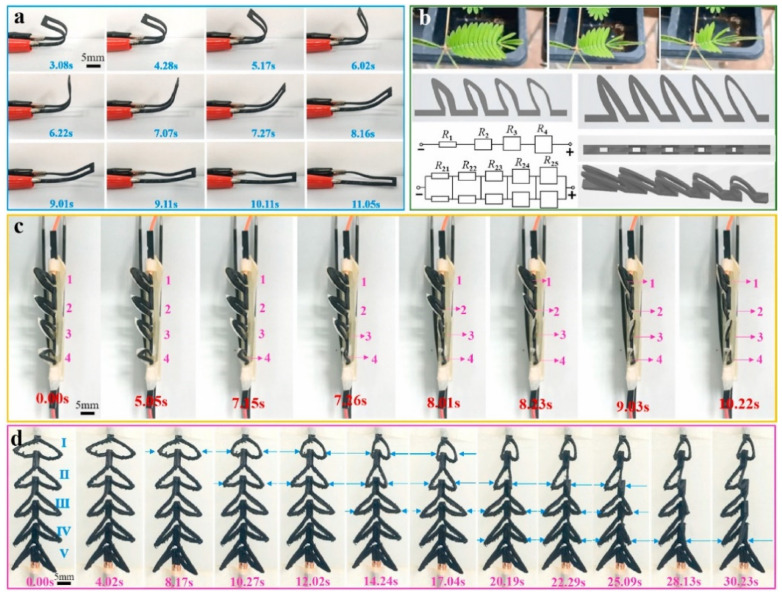
4D printing of electroactive shape-changing samples. (**a**) Shape-changing behavior when exposed to 200 V DC electrical stimuli; (**b**) Design and circuit design of biomimetic mimosa leaves; (**c**) Single-row demonstration of biomimetic mimosa leaves, and (**d**) Double-row demonstration of biomimetic mimosa leaves. Reproduced with permission from [233]. Copyright (2021) Elsevier.

In tissue engineering, stimuli responsiveness is often exploited as sensors or actuators. Grinberg et al. used FDM 4D printing to create an artificial knee prosthesis with piezoelectric properties that can behave both as mechanical bio-scaffold as well as sensors, using piezoelectric barium titanate mixed with polyamide 11 as its electroactive materials [232]. The main goal of creating an implant with piezoelectric property is so that the implant can convert the data of experienced mechanical load into electrical signals, which are then converted back again to predict the mechanical load to provide an accurate report to the medical team. When subjected to an artificial mechanical load similar to that of walking condition, the generated data are perfectly in-phase with the applied load, with good sensitivity and linearity, highlighting its potential for application as smart 4D prostheses. 4D printing’s potential as actuators were demonstrated by Chen et al. who managed to exploit its electro-responsive shape-changing properties to mimic the biological functions of various biological functions (Figure 14) [233]. Carbon black and PLA were chosen as the base materials, fabricated using FDM. Some of the actuating functions successfully demonstrated were the closing of mimosa leaves and the movement of butterfly wings, and the result of this study has the potential to be applied in biomedical applications such as muscle actuators.

## 5. Conclusions and Future Perspectives

In this review, recent advancements in CP-based electroactive scaffolds have been highlighted, showing their great potential for bone, nerve, skin, skeletal muscle, and cardiac muscle tissue engineering due to their ability to distribute ES directly to the target tissues with positive responses in promoting tissue regeneration. The review also highlighted several common weaknesses in the current generation of CP-based scaffolds including mechanical properties, biocompatibility, hydrophobicity, and biodegradability. Currently, many researchers have overcome these issues through novel strategies such as introducing double or triple networks to improve mechanical strength, adding chemical groups to improve biocompatibility, and utilizing CPs low melting point (compared with other electrically conductive fillers) to improve manufacturability. However, extensive research still needs to be carried out to translate this approach into practical uses. Future studies in CP-based electroactive scaffolds may consider the following points:

(1) Many studies have demonstrated excellent results in films and fibers architecture. However, it should be noted that the three-dimensional environment may be different, and the current challenge in faithfully mimicking the native environment of tissue to guide 3D cellular alignment still remains. Studies with films and fibers architecture should aim to use the substrate to fabricate a 3D implantable scaffold, and preferably conduct the tests up to in vivo stage.
(2) Although the proof-of-concept in fabricating biodegradable CPs based on conductive oligomers have existed, many have reported very short time of degradation (less than one week), which may not be enough time for the natural tissues to recover. Future studies may wish to consider tuning the degradation rate to better match the rate of natural tissue recovery, while also not forgetting the other important properties of an effective electroactive scaffold. This promising approach in general is still rarely explored, and an even more extensive studies should be done in this field compared to its non-biodegradable counterpart.
(3) Electroactive scaffold in itself has the ability to enhance bioactivity as it can passively provide electrical cues to the tissue microenvironment, and ES can be further used to actively enhance the rate of recovery and its effectiveness has been demonstrated numerous times. However, many studies still have not opted to take advantage of ES, possibly due to the dizzying amounts of operating parameters that need to be considered in order for the ES to be effective and not harmful to the tissues. It is encouraged for future studies to take advantage of ES and view electroactive scaffold as an active media to enhance the efficacy of ES, instead of simply relying on the conductivity of electroactive scaffold in providing passive cues.

An effective scaffold must possess not only one or two suitable properties, but must strive to achieve excellence in all of them. Many studies have reported the effect of varying CP concentration or modifying the CPs relative to the scaffold’s properties, and it is necessary to consider that we often need to compromise one property in favor of enhancing others (e.g., decreasing CP content leads to higher biocompatibility but lower conductivity, etc.). Fine-tuning of CP-to-matrix ratio, as well as exploring the possible enhancement strategies should be considered, so that all the necessary properties of the scaffold can be in the ideal range for in vivo clinical applications.

### Future Outlook: Towards Realizing In Vivo Applications of CP-Based Electroactive Scaffolds

Presently, the focus of most studies is still on the step of optimizing the scaffold’s properties. While it is important to make sure that the scaffold’s possess suitable properties, there are additional factors that must be accounted for when attempting to move towards pre-clinical animal studies, including issues such as systemic immune response, variety of cell types present, and the possible interactions of the scaffold with various biomolecules in the microenvironment that may cause the scaffold to fail in vivo regardless of how well it performs during the in vitro stage [234]. Nevertheless, the select few that managed to break through the in vitro stage barrier display very promising results, with some having significantly better performance compared to the currently available methods of treatment. For instance, Qu et al.’s work in developing degradable conductive injectable hydrogel comprising of oxidized hyaluronic acid and N-carboxyethyl chitosan functionalized with aniline tetramer and loaded with amoxicillin (D-OHA-AT/CEC) displays significantly improved wound closure rate compared to Tegaderm™ (which is regarded as the current benchmark of commercially available wound dressing), as well as ticking the boxes of being both degradable and injectable [235]. The significance of ES in conjunction with an electroactive scaffold was also highlighted by Huang et al., where the group fabricated chitosan/PPy scaffold with aligned microchannels, and then implanted it into a rat model with a large nerve defect [236]. The conductive scaffold with ES clearly outperforms the conductive scaffold without ES as well as the non-conductive scaffold, both functionally and histologically, resulting in a significantly faster rate of recovery. However, the study did not compare the scaffold/ES performance against other types of implants (i.e., autograft, xenograft), thus it is still unclear whether this method actually provides a noticeable benefit in vivo.

Thus far, the field of wound dressing and skin tissue engineering is perhaps the one that is the nearest to successful in vivo clinical applications. This is evidenced by a recent report by Lu et al. demonstrating the ability of ES/electroactive scaffold in effectively treating diabetic wounds (in which the animal model is immunocompromised, and as such is significantly more challenging than simple acute wounds) [37]. However, other applications, even nerve tissue engineering, which is historically the first one to be explored [237], are still relatively modest in terms of progress and achievements toward in vivo application. The ones that have passed into in vivo testing have incorporated and combined many strategies into one product, they have gone through a series of preliminary studies to get up to this stage, and even they are still not completely free from issues.

Although the benefit of ES in tissue engineering is clear, as evidenced by this review, research groups have found it very difficult to optimize the numerous amounts of parameters available for ES therapy (AC/DC, voltage, frequency, interval, etc.). Nevertheless, the proof of concept and benefits of ES in tissue engineering has been clearly shown in the literature by demonstrating established theories behind their working principles, and we believe that the transition to clinical use or CP-based electroactive scaffolds is only a matter of time before research successfully further optimize their design and application.

## Data Availability

Not applicable.
